# Rapid Diversification of a Natural Heterosigma akashiwo Virus Population during a Host Bloom

**DOI:** 10.1264/jsme2.ME26018

**Published:** 2026-07-11

**Authors:** Jun Xia, Lingjie Meng, Yue Fang, Hiroki Ban, Yusuke Okazaki, Takashi Yoshida, Hisashi Endo, Keizo Nagasaki, Hiroyuki Ogata

**Affiliations:** 1 Institute for Chemical Research, Kyoto University, Gokasho, Uji 611–0011, Japan; 2 Hatsukaichi Field Station, Fisheries Technology Institute, Japan Fisheries Research and Education Agency, Maruishi, Hatsukaichi, Hiroshima 739–0452, Japan; 3 Graduate School of Agriculture, Kyoto University, Kitashirakawa-Oiwake, Sakyo-ku, Kyoto 606–8502, Japan; 4 Faculty of Science and Technology, Kochi University, Nankoku, Kochi 783–8502, Japan

**Keywords:** giant virus, algal bloom, Heterosigma akashiwo virus, microdiversity, Uranouchi Bay

## Abstract

Despite the ecological importance of viruses, our understanding of their evolutionary dynamics in natural environments remains limited. This gap is particularly pronounced for giant dsDNA viruses of the phyla *Nucleocytoviricota* and *Mirusviricota*. Knowledge on their population genetic dynamics is mostly derived from a small number of laboratory-based experiments, while patterns in nature are rarely observed. To overcome this limitation, we traced the genetic structure and transcription status of Heterosigma akashiwo virus (HaV) using high-frequency, time-resolved sampling during a host bloom in a coastal area of Japan by integrating cell counting, metabarcoding, and metagenomic and metatranscriptomic sequencing. The results obtained revealed that HaV dominated the giant virus community in most samples, with relative abundance up to 56%. Despite its high abundance, the HaV population exhibited a low level of microdiversity, but had a higher pN/pS ratio than other giant viruses in the study site. Microdiversity increased during the early sampling period, peaked mid-sampling, and decreased during the later period, consistent with rapid diversification during viral expansion, which may be driven by both *in situ* mutations and the succession of pre-existing minor variants. Several accessory genes, including a glycosyltransferase and an endonuclease, were highly expressed, providing functional evidence consistent with host interaction-driven selective pressure during the bloom. Collectively, these results indicate that HaV population dynamics during algal blooms are shaped by host-driven selection acting on standing genetic variations.

Viruses generally evolve much faster than cellular organisms (Duffy *et al.*, 2008; Holmes, 2010). Among viruses, giant viruses (GVs) typically exhibit lower mutation rates than smaller viruses (*e.g.*, single-stranded DNA [ssDNA] and RNA viruses); however, they are still higher than those of cellular organisms (Sanjuán *et al.*, 2010; Duchêne and Holmes, 2018). GVs are large double-stranded DNA (dsDNA) viruses, typically in *Nucleocytoviricota* (Aylward *et al.*, 2021) and *Mirusviricota* (Gaïa *et al.*, 2023), which infect a wide range of eukaryotes (Iyer *et al.*, 2006). Their large genomes are partly due to frequent horizontal gene transfer (HGT) from cellular organisms and viruses (Irwin *et al.*, 2022; Wu *et al.*, 2024; Yi *et al.*, 2024), which enhances adaptive evolution through the acquisition of auxiliary metabolic genes modulating host cellular metabolism during infection (Ha *et al.*, 2021; Brahim Belhaouari *et al.*, 2022; Meng *et al.*, 2023b). Intra-species diversity (*i.e.*, microdiversity) may also be important for the adaptation of GVs. A recent study revealed that in the Uranouchi Inlet, marine GVs with a persistent presence across seasons generally exhibited higher levels of microdiversity than those showing a sporadic appearance, suggesting that a larger effective population size enhanced the fitness of GVs under the virus-host arms race during prolonged interactions with their hosts (Fang *et al.*, 2025). However, the short-term and high-resolution evolutionary dynamics of GVs in natural environments have yet to be characterized in detail.

Challenges are associated with tracking the short-term evolutionary dynamics of natural GV communities because the community changes more through the advection and diffusion of water masses than through viral evolution. The Uranouchi Inlet in Kochi Prefecture, Japan is an enclosed eutrophic embayment with limited freshwater inflow and restricted water exchange with offshore waters. Due to its narrow and elongated topography and weak flushing, strong stratification and seasonal hypoxia frequently develop in the bottom layer (Munekage and Kimura, 1990; Jaysankar *et al.*, 2009). Harmful algal blooms (HABs), which may be easily tracked, occur frequently in the Uranouchi Inlet (Takahashi *et al.*, 2021). Bloom events are accompanied by marked changes not only in plankton communities, but also in viral communities (Vincent *et al.*, 2023). Therefore, this inlet provides an ideal environment for observing the evolutionary dynamics of plankton and viral communities with minimal effects of water mass changes (Prodinger *et al.*, 2021; Fang *et al.*, 2025). One of the major HAB species in the Uranouchi Inlet is the raphidoflagellate *Heterosigma akashiwo*, which belongs to the family Chattonellaceae and often blooms in late spring (Prodinger *et al.*, 2021; Funaoka *et al.*, 2023).

Some GVs are known algal bloom regulators that shape microbial community structures and directly or indirectly affect the duration and intensity of blooms by killing blooming algae (Nagasaki *et al.*, 1994b; Brussaard *et al.*, 1996; Jacquet *et al.*, 2002; Gajigan *et al.*, 2025). Heterosigma akashiwo virus (HaV; family *Phycodnaviridae*, genus *Raphidovirus*, species *Raphidovirus japonicum*) is a GV that infects *H. akashiwo* and is known to play a role in terminating HAB through its lytic infection (Nagasaki *et al.*, 1994a, 2002). The sporadic boom-and-bust population dynamics of *H. akashiwo* and associated HaV make this alga-virus system an intriguing model for understanding their co-evolutionary processes. A recent cross-infection study involving 60 *H. akashiwo* strains and 22 HaV strains revealed highly diverse infection spectra, ranging from strain-specific to broad host-range types (Funaoka *et al.*, 2023), suggesting the existence of genetic diversity in both host and virus populations that have an impact on infection specificity. Rapid shifts in the viral genetic structure may emerge not only from the accumulation of *in situ* mutations, but also from the dynamic succession of pre-existing variants; specifically, minor variants that persist at low frequencies may rapidly expand and become dominant when their specific host strains proliferate (Roux *et al.*, 2017; Zhou *et al.*, 2025). Therefore, accounting for the dynamics of this standing microdiversity is crucial for a comprehensive understanding of viral adaptive processes during bloom events. However, similar to their host, HaVs show a sporadic pattern of proliferation dynamics, which may lead to a reduced level of genetic diversity due to recurrent population bottlenecks. The level of genetic diversity and the dynamics of a natural HaV population have yet to be exami­ned.

To investigate the diversity of HaV at the population level, we conducted high-frequency, daily sampling at the Uranouchi Inlet during a *H. akashiwo* bloom period. We successfully recovered a nearly complete environmental HaV genome together with other GV genomes through metagenomics. By integrating these time-resolved metagenomic (metaG) data with cell count, 18S rDNA metabarcoding (metaB), and metatranscriptomic (metaT) data, we characterized the fine-scale temporal dynamics of the genetic structure and gene expression of a HaV population in the inlet, which provide insights into the eco-evolutionary dynamics of HaV and other GVs during algal blooms.

## Materials and Methods

### Sampling

Seawater samples were collected at two sites within the Uranouchi Inlet: A-site in the red tide area of the innermost bay (133.40°E, 33.43°N) and B-site near a fishery farm located in the middle of the inlet (133.36°E, 33.41°N) (Fig. S1). Sampling at A-‍site was performed aboard the small engine-powered training vessel Triton of Kochi University, while sampling at B-site was conducted using a small electric-powered rubber boat. Sampling was performed from May 30 to June 7, 2019— consecutively on 3 days at A-site (June 3 to 5 or Day-5 to Day-7) and 9 days at B-‍site (May 30 to June 7; Day-1 to Day-9) (Table S1). Sampling was conducted during the daytime (08:20–13:50). Samples at A-‍site were collected from clearly observable *H. akashiwo* blooming patches.

Surface and subsurface chlo­rophyll maximum (SCM; 2.5–3.1 m) layers were sampled at A-site, while SCM (1.0–3.1 m) samples were collected at B-site. Seawater was prefiltered through a 144-μm mesh to remove large particles. In each metaG sample targeting the detection of viral genomes from free virions, 10 L of seawater was collected in a sealed flexible plastic container and transported on ice to a nearby coastal laboratory (approximately 60 min from A-site and 15 min from B-site). Samples were then filtered sequentially through polycarbonate membrane filters with a pore size of 3 μm and a diameter of 142 mm (Nuclepore, Whatman) and four 0.22-μm Sterivex sterile filter units (Millipore) on board (three 3- to 144-μm and fourteen 0.22- to 3-μm samples were used in the downstream anal­ysis). In each metaT sample targeting the detection of viral transcripts within the host cells, 500–1,000 mL seawater was filtered through polycarbonate membrane filters with a pore size of 3 μm and a diameter of 47 mm (Nuclepore, Whatman). After filtration, membrane filters were aseptically removed using sterilized forceps, folded, and placed into sterile 1.5-mL microcentrifuge tubes, adding 1.5 mL RNAlater solution (Thermo Fisher Scientific) for preservation, upon arrival at the coastal laboratory, and subsequently transported on dry ice within 2 days to a −80°C freezer for long-term storage prior to RNA extraction (nine 3- to 144-μm samples were used in the downstream anal­ysis). In each 18S rDNA metaB sample, 500–1,000 mL of seawater was filtered through polycarbonate membrane filters with a pore size of 3 μm and a diameter of 47 mm (Nuclepore, Whatman) immediately on board (fourteen 3- to 144-‍μm samples were used in the downstream anal­ysis). Filters were transported on ice to the coastal laboratory, stored at –20°C, and then transferred on dry ice within 2 days to –80°C storage until DNA extraction. Regarding microscopic cell counts, 2 L of unfiltered surface or SCM seawater was fixed with 2% (v/v) Lugol’s iodine solution on board following the recorded phytoplankton preservation protocols (Karlson *et al.*, 2010), and transported on ice to the coastal laboratory within 15–60 min, where samples were stored at 4°C until analyzed.

### DNA and RNA extraction

DNA and RNA were extracted following previously described protocols (Endo *et al.*, 2018). Purified DNA was dissolved in 30 μL of low-TE buffer for the metaB anal­ysis and in 30 μL of Milli-Q ultrapure water for the metaG anal­ysis. Regarding RNA extraction, samples were mixed with β-mercaptoethanol, RLT lysis buffer (QIAGEN), and glass beads, followed by bead beating and centrifugation. The supernatant was processed using the RNeasy Mini Kit (QIAGEN) following the manufacturer’s instructions. Residual genomic DNA was removed by on-column DNase digestion using the RNase-Free DNase Set (QIAGEN), in which 80 μL of the DNase incubation mix was applied directly onto the membrane and incubated at room temperature for 15 min, followed by washing with RW1 buffer prior to RNA elution. Extracted DNA and RNA were stored at −20°C for later use. DNA and RNA concentrations and purities (A260/280) were quantified using a Qubit fluorometer (Thermo Fisher Scientific), Nanodrop spectrophotometer (Thermo Fisher Scientific), and TapeStation system (Agilent Technologies).

### Library construction, sequencing, and data processing

MetaG libraries were prepared using the TruSeq Nano DNA Kit and sequenced on the Illumina MiSeq 300PE platform.

MetaT libraries were prepared using the NEBNext^®^ Poly(A) mRNA Magnetic Isolation Module, NEBNext^®^ UltraTMII Directional RNA Library Prep Kit, and NovaSeq 6000 150PE sequencing was conducted.

In the metaB anal­ysis, the V8–V9 regions of the eukaryotic 18S rDNA gene were amplified using the primers V8f–1510r (positions 1,422–1,797 in the *Saccharomyces cerevisiae* gene) following a previously reported method (Bradley *et al.*, 2016; Prodinger *et al.*, 2020). PCR products were checked by agarose gel electrophoresis, purified, and diluted with 25 μL of ultrapure water. MetaB libraries were constructed using MiSeq Reagent Kit v2 and sequenced on the Illumina MiSeq 300PE platform. MetaB reads were processed with QIIME2 (v2020.2) (Bolyen *et al.*, 2019). Trimming, quality filtering, denoising, merging, dereplication, chimera removal, and the generation of amplicon sequence variants (ASVs) were performed using the DADA2 plugin integrated in QIIME2 (q2-dada2 version 2020.2.0) (Callahan *et al.*, 2016). Taxonomic classification was performed using the SILVA 138 database (Quast *et al.*, 2013) and PR2 database (version 4.14.0) (Guillou *et al.*, 2013) with BLASTn (≥97% identity). After excluding ASVs classified as metazoa or fungi as well as singleton ASVs, sequence data were subsampled to the minimum size among all 14 samples to compute the relative abundances of ASVs.

After quality filtering, sequencing yielded 2.09 million metaB paired reads (1.26 Gbp, *n*=14), 2.56 billion metaG reads (725.75 Gbp, *n*=17), and 0.65 billion metaT reads (195.21 Gbp, *n*=9) (Table S4).

### Reconstruction of GV-MAGs

The pipeline hedera (version 0.0.5) (https://github.com/banhbio/hedera) developed in previous studies (Fang *et al.*, 2025; Liu *et al.*, 2025) was improved for the generation of GV metagenome-assembled genomes (GV-MAGs). The complete workflow from raw read processing to the final GV-MAG construction is described and illustrated in Supplementary Text and Fig. S2. Briefly, after the co-assembly of metaG reads from 17 samples (three 3- to 144-μm samples and fourteen 0.22- to 3-μm samples), 2,515,850 contigs (≥1 kbp, mean length 2,796 bp) were generated, of which 590,170 contigs (≥2.5 kbp, mean length 6,910 bp) were‍ ‍retained for binning. Raw bins (1,794 bins) produced by MetaBAT2 using differential coverage across all 17 metaG samples, as well as 1,734 unbinned contigs (≥30 kbp) were included in the downstream anal­ysis. Based on the GV marker gene density index (>5.75) (Fang *et al.*, 2025), 432 bins and 65 single contigs were retained. None were removed during the GV contig assessment. After delineation and second decontamination using Anvi’o (Eren *et al.*, 2015), 504 GV-MAG candidates were retained. One contig was later removed from a bin due to the presence of abundant prokaryotic core genes. Subsequent manual delineation, a phylogeny-informed MAG assessment (PIMA), and dereplication (no MAG was duplicated at the 95% ANI threshold) yielded 424 high-quality GV-MAGs (59.3 kbp–1.36‍ ‍Mbp), including a MAG corresponding to a HaV population.

The additional manual curation and refinement of HaV-MAG were conducted. All contigs obtained from the metaG co-assembly were aligned to the reference genome of the isolate HaV53 (Accession: NC_038553.1) (Ogura *et al.*, 2016) using BLASTn (best hit, E-value<1e-5). Contigs showing reliable alignments were identified as potential HaV-contigs. Reads mapping to these contigs were extracted from sorted BAM files with Samtools (Li *et al.*, 2009) and then reassembled to final HaV-contigs using SPAdes (v3.15.4) (Nurk *et al.*, 2017) with the “--meta” option. The reassembled HaV-contigs were reordered to form the final HaV-MAG based on the HaV53 reference genome by GenomeNet’s ReCCO (v1.0-beta) (https://www.genome.jp/ftp/tools/ReCCO/ReCCO_Readme.html). The original automatically generated HaV-MAG was replaced with this refined MAG in subsequent anal­yses.

### Sequence comparison, phylogeny, and function anal­ysis

The basic statistics of MAGs were obtained using SeqKit (v2.3.0) (Shen *et al.*, 2016). Average nucleotide identity (ANI) between genomes was calculated with FastANI (v1.33) (Jain *et al.*, 2018). Dereplication at 95% ANI was performed among the GV-‍MAGs generated in the present study and genomic data in the GOEV database (Gaïa *et al.*, 2023) using dRep (v3.4.2) (dRep dereplicate options: -sa 0.95 -l 1000 -comp 50 --S_algorithm ANImf -nc 0.5 -N50W 0 -sizeW 1 --ignoreGenomeQuality) (Olm *et al.*, 2017). Amino acid identity between HaV-MAG and HaV53 was assessed by BLASTp (E-value<1e-3).

A maximum-likelihood phylogeny of nucleocytoviruses (nucleocytoplasmic large DNA viruses [NCLDVs]) was reconstructed using IQ-TREE2 (v2.2.2.6) (Minh *et al.*, 2020) with the LG+I+F+G4 model based on a concatenated alignment of seven conserved markers (DEAD/SNF2-like helicase [SFII], DNA-directed RNA polymerase alpha subunit [RNAPL], DNA polymerase family B [PolB], Transcription initiation factor IIB [TFIIB], DNA topoisomerase II [TopoII], Packaging ATPase [A32], and Poxvirus Late Transcription Factor [VLTF3]) generated by ncldv_markersearch.py (v1.1) (Aylward *et al.*, 2021). Mirusvirus major capsid protein HK97 sequences were identified with hmmsearch (E-value<1e-5) (v3.4) (Finn *et al.*, 2011), aligned with MAFFT (v7.525) (Katoh and Standley, 2013), trimmed by trimAl (v1.5.0, “-gt 0.1”) (Capella-Gutiérrez *et al.*, 2009), and used to construct a maximum-likelihood tree using IQ-TREE2 under the LG+R6 model. All trees were visualized in Anvi’o (v7.1) (Eren *et al.*, 2015). Relative evolutionary divergence (RED) values were calculated to identify the family- and order-level clades of GVs.

Gene-level synteny between HaV-MAG and the HaV53 reference genome was assessed by DiGAlign (a similarity search using BLASTn, E-value<1e-2 is shown in green colors) (v2.0) (Nishimura *et al.*, 2024). The HaV-MAG genomic structure was visualized using the R package gggenes based on GFF annotations. Multiple sequence alignment of the HaV major capsid protein was performed using Clustal Omega (v1.2.4) (Sievers *et al.*, 2011). Protein 3D structures were predicted using AlphaFold3 (Abramson *et al.*, 2024) via the AlphaFold Server and rendered in ChimeraX (v1.9) (Meng *et al.*, 2023a).

GV ORFs predicted by prodigal were annotated by hmmsearch (E-value<1e-5) against GVOG (Aylward *et al.*, 2021), 149 core NCVOGs (Yutin *et al.*, 2009; Gaïa *et al.*, 2023), EggNOG (euk+arc+bac+vir) (Hernandez-Plaza *et al.*, 2023), and Pfam-A (Mistry *et al.*, 2021). Annotation priority was as follows: (I) GVOG annotated by NCVOG; (II) GVOG annotated by EggNOG; (III) GVOG annotated by Pfam; (IV) 149 NCVOG annotation; (V) EggNOG annotation; and (VI) Pfam-A annotation. If a higher-priority annotation was labeled as “unknown”, “unannotated”, “NA”, blank, or equivalent, the next level of annotation was adopted.

### Community and population anal­yses

Qualified metaG reads from each sample were mapped to 2,241 GV genomes (424 GV-MAGs from this study and 1,817 GOEV genomes) using minimap2 (Li, 2018) in CoverM (v0.7.0) (Aroney *et al.*, 2025) with thresholds of >95% identity and >75% length of reads. The relative abundance of GV communities was estimated as transcripts per million (TPM) and normalized to 100% per sample.

Diversity indices (Simpson, Shannon, and Pielou’s J) and Bray–Curtis dissimilarities were calculated using the vegan R package. ANOSIM and Mantel tests were performed with vegan and ade4, respectively. Non-metric multidimensional scaling (NMDS) was conducted using monoMDS (k=3), and the results obtained were visualized with ggplot2.

In the microdiversity anal­ysis, simple repeats in GV-MAGs were masked (replaced with “N”) using RepeatMasker (v4.1.5) to avoid the inclusion of ambiguously mapped reads (Tarailo-Graovac and Chen, 2009). Qualified metaG reads from each sample were mapped to repeat-masked GV-MAGs with bowtie2 (v2.5.0) (Langmead and Salzberg, 2012). The average depth for each gene was calculated based on sorted BAM files using samtools (Li *et al.*, 2009). Microdiversity, including single nucleotide variants (SNVs), nucleotide diversity (ND), and pN/pS, was assessed for each MAG in every sample using inStrain (v1.5.1) (Olm *et al.*, 2021) with the “-f 0.01” parameter, based on the genes of repeat-masked GV-MAGs. ORFs with an average depth≥10 and at least 25 SNVs were retained for the anal­ysis of pN/pS ratios.

Additional ND, which is denoted as Nei’s π in this study, and the fixation index (F_ST_) for HaV-MAG were also calculated for B-site samples (depth ≥10, repeats masked) using previously described methods (Sjöqvist *et al.*, 2021; Fang *et al.*, 2025) instead of inStrain. We used Nei’s π and F_ST_ for the comparison across samples because these indices better control the nucleotide sites being compared irrespective of mapping depth differences across samples.

### MetaT anal­ysis

MetaT reads (*n*=9, 3- to 144-μm fraction) were quality-filtered by fastp (v0.23.4) (Chen *et al.*, 2018). Ribosomal RNA sequences were removed by searching qualified paired reads against the SILVA 138.1 database (Quast *et al.*, 2013) (LSURef_NR99, SSURef_NR99, RF00001 and RF00002) using SortMeRNA (v4.3.6) (Kopylova *et al.*, 2012). TPM was calculated for each sample with CoverM (Aroney *et al.*, 2025) against the 424 GV-‍MAGs derived from metaG data. TPM values were normalized to 100% per sample to represent relative expression abundance.

### Accession numbers

All reads generated in the present study were deposited to the DNA Data Bank of Japan (DDBJ) under the bioproject PRJDB37393, with biosample numbers SAMD01619950 to SAMD01619977.

### Data availability

GV MAGs generated in the present study and related data files (424 GV-MAGs genome files, gene files, phylogenetic tree files, hmm profiles, and a summarized table for inStrain output) are‍ ‍accessed on GenomeNet at https://www.genome.jp/ftp/db/community/Uranouchi_GVMAGs_9days/.

## Results

### Abiotic and biotic conditions during the *H. akashiwo* bloom

*H. akashiwo* blooms frequently recur in spring in the Uranouchi Inlet. Therefore, routine daily sampling was conducted at B-site, a 9-day monitoring location situated near aquaculture facilities where nutrient-rich conditions often promote red tide development. A conspicuous red tide patch was visually observed at the surface of A-site on Day-5, indicating the bloom peak, with a rapid decline by Day-7. In response, additional samples targeting this spatially patchy bloom were collected around A-site while daily time-series observations at B-site were maintained.

*H. akashiwo* was dominant in samples collected at A-site (A_Day5_S, 8,464 cells mL^–1^, 53.5%; A_Day6_S, 20,611 cells mL^–1^, 65.7%), while cell densities remained low at B-site (average 58 cells mL^–1^) (Fig. 1A, Table S3). Consistent with cell counting results, *H. akashiwo*, represented by seven ASVs (ASV0580–ASV0586, among which ASV0580 dominated across all 14 samples, accounting for 94.6% of total *H. akashiwo* amplicon reads), dominated the micro­eukaryote communities in Day-5 and Day-6 samples at A-site (48.8–81.5%) (Fig. 1A). The relative abundance of *H. akashiwo* decreased on Day-7 at A-site (surface 11.0%, SCM 17.9%), while it was continuously low (<3%) in B-site samples. Members of the diatom class Mediophyceae, the dinoflagellate class Dinophyceae, and symbiotic dinoflagellates order Syndiniales were abundant at B-site (Fig. 1B). It is important to note that the renaming of Mediophyceae to Thalassiosirophyceae has recently been proposed (Kociolek *et al.*, 2026).

Eukaryotic community structures significantly differed between A-site (*n*=5) and B-site (*n*=9) (ANOSIM, *r*=0.934, *P*<0.001) (Fig. 2A). Community structures showed large differences across A-site samples, except for A_Day5_S and A_Day6_S, which were collected from the bloom patch and displayed highly similar community compositions. At B-site, community structures gradually changed in samples collected from Day-1 to Day-9.

### GV communities

The relative abundance of GV communities (for GV-MAG information, see Supplementary Figures and Text) was dominated by *Imitervirales* (52.8% on average) in most samples, followed by *Algavirales* (28.4% on average) (Fig. S3). However, at the MAG level, the HaV-MAG of the order *Algavirales* was the most abundant in 10 samples (Fig. 1C, Table S6). In contrast to the high abundance of *H. akashiwo* in samples A_Day5_S and A_Day6_S, HaV-MAG remained at a low relative abundance (<3%) in both samples. In the remaining 12 samples (3 A-site samples and 9 B-site samples), the relative abundance of HaV-MAG varied from 5.0% to 56.6% (24.8% on average). Besides HaV-MAG, one *Asfuvirales* MAG (Uranouchi_MAG_sc_580) showed high relative abundance (7.2% on average), whereas no other MAGs exceeded an average relative abundance of 3%. Only six GV-MAGs contributed more than 1% to the total viral community in at least one of 14 samples. Consistent with the high relative abundance of HaV in the samples A_Day6_SCM, A_Day7_S, and B_Day3_SCM, Shannon’s diversity and evenness of GV communities were low in these samples (Fig. S4B, Table S8).

GV community structures correlated with eukaryote community structures in B-site samples spanning 9 days (the Mantel test, Pearson’s *r*=0.471, *P*<0.05), but not in A-site samples spanning 3 days (*r*=0.076, *P*>0.05). No significant separation was observed in GV community structures between A-site (*n*=5) and B-site (*n*=9) (Fig. 2B).

### Genomic characteristics of environmental HaV

The HaV-MAG generated in the present study was composed of seven contigs (258,302 bp in total), encoding 292 ORFs and displaying a GC content of 30.0%. In comparisons with the reference HaV53 genome (274,793 bp, 30.4% GC content), HaV-MAG was 16‍ ‍kb shorter (Table 1). We defined ORFs as shared between the two HaV genomes when a ORF showed a BLASTp best hit (E-value<1e-3) from at least one ORF from the other genome. ORFs that did not pass this criterion were operationally denoted as ORFs specific to one of the genomes. As a result, 283 (96.9%) ORFs from HaV-MAG and 272 (93.2%) ORFs from HaV53 were found to be shared between these genomes (Table S10). A high level of collinearity and wide coverage were also observed in Fig. S5. Some of the ORFs in HaV-MAG were incomplete when located at the edge of contigs.

HaV-MAG displayed the full set of core genes conserved in HaV53. Six of the nine nucleocytovirus core genes (A32, MCP, PolB, TFIIB, TopoII, and VLTF-3) were detected in HaV-MAG, along with additional conserved genes, such as TFIIS, VLTF-2, and PCNA (Table S10). Regarding predicted functions, glycosyltransferases (6 ORFs) were the most numerous, followed by ring finger proteins (5 ORFs), mRNA capping enzymes (4 ORFs), KilA-N domain proteins (4 ORFs), and integrases/resolvases (4 ORFs) (Fig. S6, Table S10).

A few differences were observed in two genomes (Fig. S5). Most of the ORFs specific to HaV53 (20 ORFs) and HaV-MAG (9 ORFs) were not functionally annotated, except for transposases (3 in HaV53 and 1 in HaV-MAG), a chaperone of endosialidase (HaV53), and a helix-turn-helix XRE family-like protein (HaV53) (Table S9). These specific ORFs were mainly located in the 214.5–227.0 kbp region of the HaV53 genome, which was missing or largely different from HaV-MAG, as well as in the first contig of HaV-MAG, part of which was missing in HaV53 (Fig. S5). Although the chaperone of endosialidase (peptidase S74, pfam13884) in HaV53 (HaV53_86, 444 aa) was defined as being specific to HaV53 by our initial anal­ysis criterion, HaV-MAG was found to harbor a longer version of the orthologous gene (HaVMAG_contig_3_1, 1,529 aa) with a peptidase S74 domain (CD-search E-value: 6.36×10^–4^) and an additional large filamentous haemagglutinin (FhaB) domain (CD-search E-value: 1.40×10^–5^). FhaB is a large exoprotein domain involved in heme utilization or adhesion and was missing in HaV53_86 (Fig. S7). Another region unique to HaV53 corresponded to a repetitive coding region, predicted to encode a huge β-helical structure within an uncharacterized protein (Fig. S8) (HaV53_253: 3,663 aa; HaV-MAG_contig_6_1: 2,493 aa).

### Population structures of HaV and other GVs

We calculated the number of SNV sites for each GV-MAG using the sample in which GV-MAG displayed its maximum relative abundance. HaV-MAG exhibited a large number of SNVs (3,339, in sample B_Day3_SCM). However, the nucleotide diversity of HaV-MAG was lower‍ ‍(1.52×10^–3^) than those of other GV-MAGs showing a similar number of SNV sites (Fig. 3A).

We then calculated the pN/pS ratio for each ORF in GV-MAGs (Fig. 3B). pN/pS showed a large variance for genes with a small number of synonymous SNVs (SNV_S) (*e.g.*, SNV_S<20), while it was more stable for ORFs with a larger number of SNV_S. This result may be in part due to a stochastic effect for genes with a small number of SNVs. A small number of synonymous substitutions results in a highly unstable denominator, thereby leading to large variance in the pN/pS ratio. Therefore, for a small number of SNVs, the apparent elevation in pN/pS may not reflect the result of selection. Nonetheless, there was still a notable case. The ORF exhibiting the highest pN/pS ratio (1.477) in HaV-MAG was HaVMAG_contig_3_89, which encodes a GDP-mannose 4,6 dehydratase. In this case, the ORF showed 3 synonymous SNVs and 19 nonsynonymous SNVs, which implies positive selection acting on this gene. To ensure the reliable estimation of pN/pS ratios, we focused on ORFs with SNV_S≥25 to capture the functional constraints and purifying selection pressure in these genomes. ORFs from HaV-MAG (7 ORFs) showed a pN/pS ratio of 0.394 on average (Fig. 3C). We then selected 28 other GV-MAGs encoding at least five ORFs with SNV_S≥25 and calculated the average pN/pS ratio for each of the GV-MAGs. Of the 29 GV-MAGs, HaV-MAG displayed the highest average pN/pS ratio (Fig. 3C). The predicted functions of the seven ORFs for which pN/pS ratios were computed included a C-type lectin, a chaperone of endosialidase, a glutathione-dependent formaldehyde-activating enzyme, three helix-turn-helix domain (HTH)-containing proteins, and a protein of unknown function (Table S11).

### Population dynamics of HaV and other GVs

During the 9-days sampling period at B-site, the nucleotide diversity of the HaV population represented by HaV-MAG displayed three phases with lower nucleotide diversity than previous data on *Algalvirales* MAGs (Fang *et al.*, 2025): (i) a gradual increase from Day-1 to Day-3; (ii) a plateau from Day-3 to Day-7; (iii) a decrease from Day-7 to‍ ‍Day-9 (Fig. S9A). Nucleotide diversity levels did not correlate with the relative abundance of HaV-MAG or its host (the 18S relative abundance and cell density of *H. akashiwo*) (Pearson’s correlation test, *P*>0.05).

Genetic differences between HaV-MAG populations in different samples were estimated using F_ST_ (253,476 comparable sites, covering 98.1% HaV-MAG). Day-2 and Day-9 populations showed large genetic distances from other samples (F_ST_ on the order of 10^–4^), whereas pairwise F_ST_ values remained consistently low from Day-3 to Day-7 (on the order of 10^–5^) (Fig. S9B and C).

### Transcription landscape of HaV

At B-site, the expression levels of HaV-MAG ORFs were high within the GV communities from Day-1 to Day-8 (>3%) (Fig. 4A). Across the 9 days, the highest expression levels were generally observed in a *Pandoravirales* MAG (Uranouchi_GVMAG_808). HaV-MAG showed the second-highest average expression level overall; however, it reached a maximum of 33% on Day 3 and became the most highly expressed MAG. The relative transcription level of HaV-MAG correlated with the relative abundance of HaV-MAG (Pearson’s *r*=0.687, *P*<0.05) and the 18S rDNA abundance of the host *H. akashiwo* (Pearson’s *r*=0.945, *P*<0.001).

The most highly transcribed ORF encoded a glycosyltransferase (<50% amino acid identity to any sequence in the nr database), followed by an endonuclease belonging to a staphylococcal nuclease homologue (PF00565) (Fig. 4B). Although the six core marker genes of HaV-MAG were expressed from Day-1 to Day-8, their average expression levels (0.01 to 0.22% per gene) were markedly lower than the ten most expressed ORFs (1.46 to 15.53% per gene). The expression patterns of HaV-MAG changed with time, particularly from Day-6 to Day-9 (Fig. S10).

## Discussion

Phytoplankton blooms are short-lived, but high-impact events that restructure algal communities and impose strong selection on both hosts and viruses. However, how viral populations, particularly those of GVs, evolve over the short time scales of natural blooms remains unclear. *H. akashiwo* blooms often occur in late-spring and early-summer seawater (Anderson *et al.*, 2002; Dursun *et al.*, 2016) and have been extensively exami­ned in the Uranouchi Inlet. In the present study, our 9-day sampling campaign successfully captured a *H. akashiwo* bloom at two contrasting sites: an innermost site (A-site) dominated by *H. akashiwo* and a site near a fishery farm (B-site) dominated by diatoms and dinoflagellates. Therefore, this natural setting provided a unique opportunity to track the population dynamics of HaV *in situ*.

HaV was an abundant virus throughout this period at both A-site and B-site (Fig. 1C). It persisted at a high relative abundance even when host densities were low (*e.g.*, at B-‍site) (Fig. 1A and B), while low viral abundance was observed in samples with high host densities (A_Day5_S and A_Day6_S). This temporal and spatial decoupling may be explained by HaV’s long latent period (30–33 h) and large burst size, which may delay viral accumulation relative to host proliferation and sustain high viral levels after host decline (Nagasaki *et al.*, 1999). A similar pattern was also reported during algal blooms in other systems (Hevroni *et al.*, 2023). HaV rapidly dominated GV communities following the bloom of the host population at the study site, as previously demonstrated in other coastal areas (Tomaru *et al.*, 2004). In addition to this “time lag”, the decoupling pattern may reflect the spatial patchiness and short-lived nature of bloom events in the inlet, as well as the hydrodynamic transport of virus particles or infected cells between sites. In addition, vertical decoupling between host and virus distributions may have occurred because samples were collected from the SCM layer, while host cells may have been concentrated in surface waters during bloom development or transported to deeper layers following bloom decline. Therefore, the abrupt increase in HaV is interpreted as being driven by host-specific dynamics and hydrographic processes, rather than as a consequence of a community-wide change.

The HaV population harbored many SNVs; however, its nucleotide diversity was lower than those of other GV-MAGs with similar numbers of SNVs (Fig. 3A). This result indicates that many SNV sites in HaV-MAG were dominated by major alleles. This pattern is consistent with a genetic bottleneck linked to boom-and-bust host dynamics or the founder effect driven by hydrography, whereby a limited number of viral genotypes initiated infection at the onset of the host bloom, followed by explosive population expansion (Zwart and Elena, 2015). During rapid expansion, new mutations may have accumulated faster than selection was able to efficiently filter, potentially inflating pN/pS by allowing slightly deleterious variants to transiently persist. Furthermore, this diversification may not rely solely on *de novo* mutations; it may also reflect the rapid succession of pre-existing minor variants. Within a heterogeneous host bloom, multiple HaV genotypes that previously persisted below the detection limit may have undergone explosive proliferation upon encountering their specifically compatible host strains. These minor variants may in part have contributed to the observed microdiversity patterns. This interpretation is supported by the result showing that HaV exhibited higher average pN/pS ratios than other GVs for ORFs with reliable pN/pS estimates (Fig. 3B and C). However, we cannot exclude the alternative possibility that these elevated pN/pS values resulted from positive selection acting on specific sequence regions of the genes used for the pN/pS calculation.

The temporal dynamics of the HaV population structure further support such a view of genetic drift. The nucleotide diversity of the HaV population increased during the early phase of the sampling period, which may reflect either the ongoing generation of genetic diversity during viral proliferation or the simultaneous expansion of pre-existing minor viral genotypes from external or cryptic sources (such as sediment). Diversity subsequently decreased toward the end of the sampling period (Fig. S9A). This decline in viral microdiversity suggests a limited number of successful genotypes infecting the available host. Consistent with this interpretation, 18S rRNA amplicon data indicated that the *H. akashiwo* population was overwhelmingly dominated by a single ASV across all 14 samples. This stable host population structure may have contributed to the observed contraction (*i.e.*, selection) in viral microdiversity. However, because it is difficult to ensure that identical water masses were sampled throughout the time series, these fine-scale dynamics need to be interpreted with caution.

At the functional level, several results indicated that the host-virus interaction was the critical factor shaping the HaV population during rapid population expansion. The transcriptomic anal­ysis showed that a HaV-specific glycosyltransferase was expressed at markedly higher levels than core genes (Fig. 4B), in contrast to previous findings in which GV transcription was strongly biased toward core genes (Ha *et al.*, 2021). GTs encoded by GV are known to modify viral or host glycoproteins by manipulating sugar residues, thereby facilitating host recognition, attachment, and immune evasion (Speciale *et al.*, 2022). The HaV GT belongs to the non-Leloir GT-C fold (Mestrom *et al.*, 2019) and contains multiple predicted transmembrane α-helices (Fig. S11), indicating its involvement in the glycosylation of viral capsid or surface-associated proteins. Therefore, the high expression of this GT may be associated with enhanced infection processes in HaV, potentially reflecting a role in host–virus interaction dynamics. Infection specificity in the HaV–*H. akashiwo* system has been linked to strain-dependent differences in viral adsorption to host cells (Funaoka *et al.*, 2023).

An additional perspective on rapid diversification at the host-virus interface is provided by gene gain and loss (Table S9). Transposases, which serve as the key enzymes mediating DNA excision and transfer (Sun *et al.*, 2015), were found to be absent from HaV-MAG, but present in the reference HaV53 genome. Although we were unable to confirm whether divergent transposases originated from a single transposable element, their heterogeneity suggests variable patterns of DNA fragment transfer during interactions with diverse *H. akashiwo* strains. In addition, the viral chaperone of endosialidase, which is essential for the assembly and folding of endosialidases that degrade surface sialic acids, was used by hosts for pathogen recognition (Schwarzer *et al.*, 2007; Tiralongo, 2010). Specifically, the orthologous gene in HaV-MAG (HaVMAG_contig_3_1, 1,529 aa) contains an additional FhaB domain that is absent in HaV53_86 (444 aa), suggesting a “domain gain” or “loss” event in HaV genomes during population evolution (Table S9 and S10, Fig. S7). Divergence in these surface- and interaction-related genes may modulate infectivity toward hosts exhibiting distinct extracellular defense traits. Collectively, these results suggest that gene gain and loss contribute to maintaining a selective advantage in the HaV-host arms race.

The present study is the first to resolve the genetic structure, population dynamics, and transcriptional activity of a natural HaV population. Although the genetic diversity of the HaV population was low, it transiently increased during the proliferation period associated with the host bloom. This dynamic genetic shift suggests that the HaV population rapidly adapts to host blooms through a combination of *in situ* evolution and the selection of standing genetic variations. In parallel, environmental HaV genomes differ in gene content from the reference HaV53 genome, indicating the genomic plasticity of HaV in natural populations. Functionally, HaV exhibits the low expression of core genes, but the high expression of accessory genes involved in host-specific interactions or the evasion of host defenses. Taken together, these results support host–virus interactions acting as purifying selective pressure on HaV during the bloom.

## Acknowledgements

This study was supported by JSPS/KAKENHI (nos. 18H02279 and 19H05667 to Hiroyuki Ogata, 17H03850 and 21H05057 to Takashi Yoshida and Hiroyuki Ogata, and nos. 19K15895 and 23K23685 to Hisashi Endo), Scientific Research on Innovative Areas from the Ministry of Education, Culture, Science, Sports and Technology (MEXT) of Japan (nos. 16H06429, 16K21723, and 16H06437 to Hiroyuki Ogata), JST PRESTO (no. JPMJPR23G3 to Hisashi Endo), and the Collaborative Research Program of the Institute for Chemical Research, Kyoto University (grant numbers 2016-28 and 2019-35). We would like to express our sincere gratitude to the staff of the SuperComputer System, Institute for Chemical Research, Kyoto University. We thank Kochi Prefecture Fisheries Promotion Department for sampling permission and providing information on the algal bloom area, weather, and hydrological characteristics. We thank Takafumi Kataoka and Shintaro Takao for chemical and nutrient anal­yses. We would also like to thank to Florian Prodinger, Chi-Yu Shih, Wenwen Liu, Tatsuhiro Isozaki, Hiroaki Takebe, Kento Tominaga, and Yoshiaki Sato for their kind instructions in both dry-lab and wet-lab experiments.

### Conflicts of interest

The authors declare that there are no conflicts of interest.

## Citation

Xia, J., Meng, L., Fang, Y., Ban, H., Okazaki, Y., Yoshida, T., et al. (2026) Rapid Diversification of a Natural Heterosigma akashiwo Virus Population during a Host Bloom. *Microbes Environ ***41**: ME26018.

https://doi.org/10.1264/jsme2.ME26018

## Supplementary Material

Supplementary Material 1

Supplementary Material 2

## Figures and Tables

**Fig. 1.**
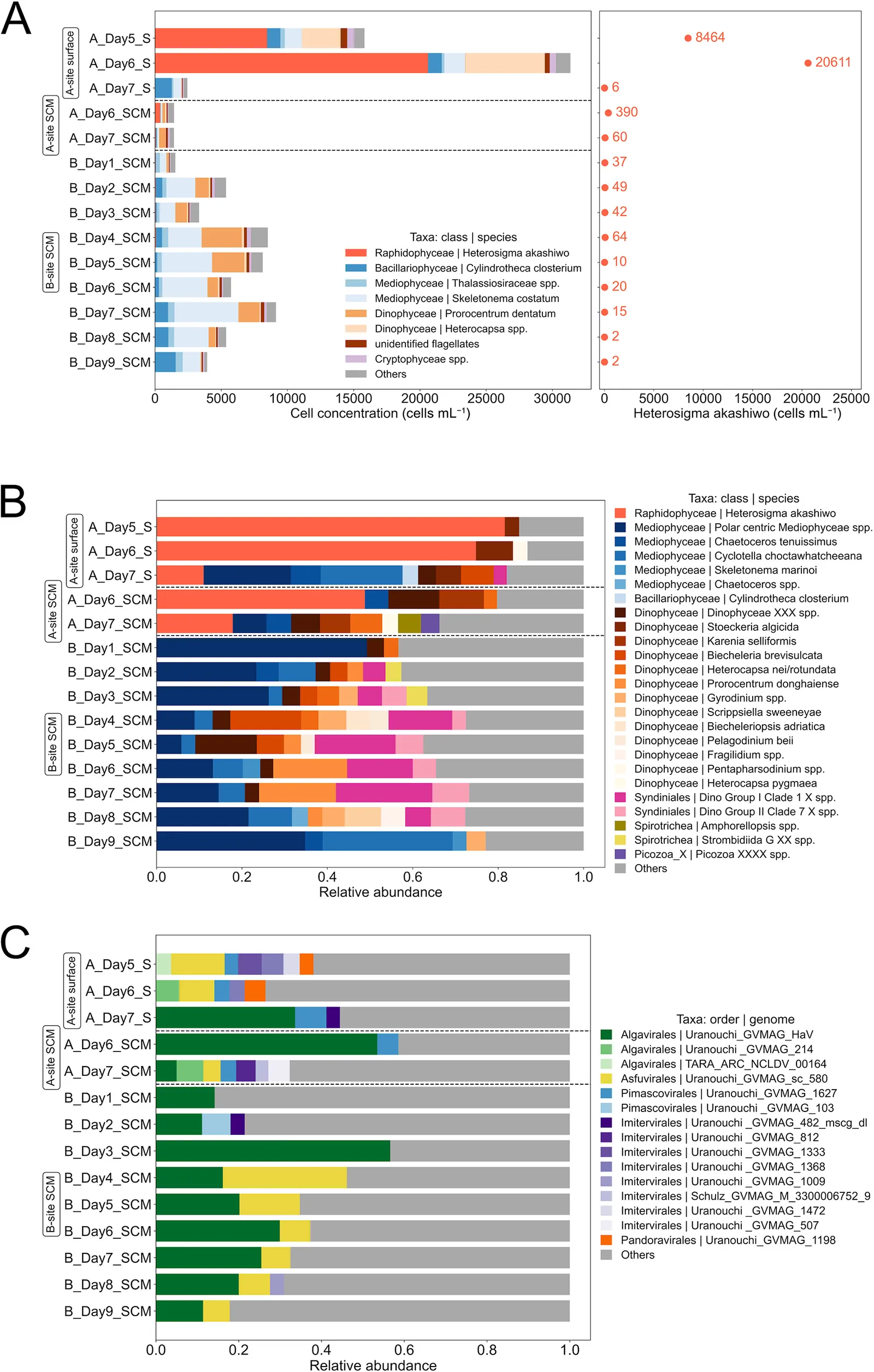
(A) Left: Mean cell counts of phytoplankton (taxa with >100 cells mL^–1^); right: cell counts of *Heterosigma akashiwo*; (B) relative abundance of the microbial eukaryotic 18S community (metazoa and fungi removed, taxa with relative abundance >3% in each sample are shown); (C) relative abundance of the giant virus community (genome relative abundance >3% in each sample is shown).

**Fig. 2.**
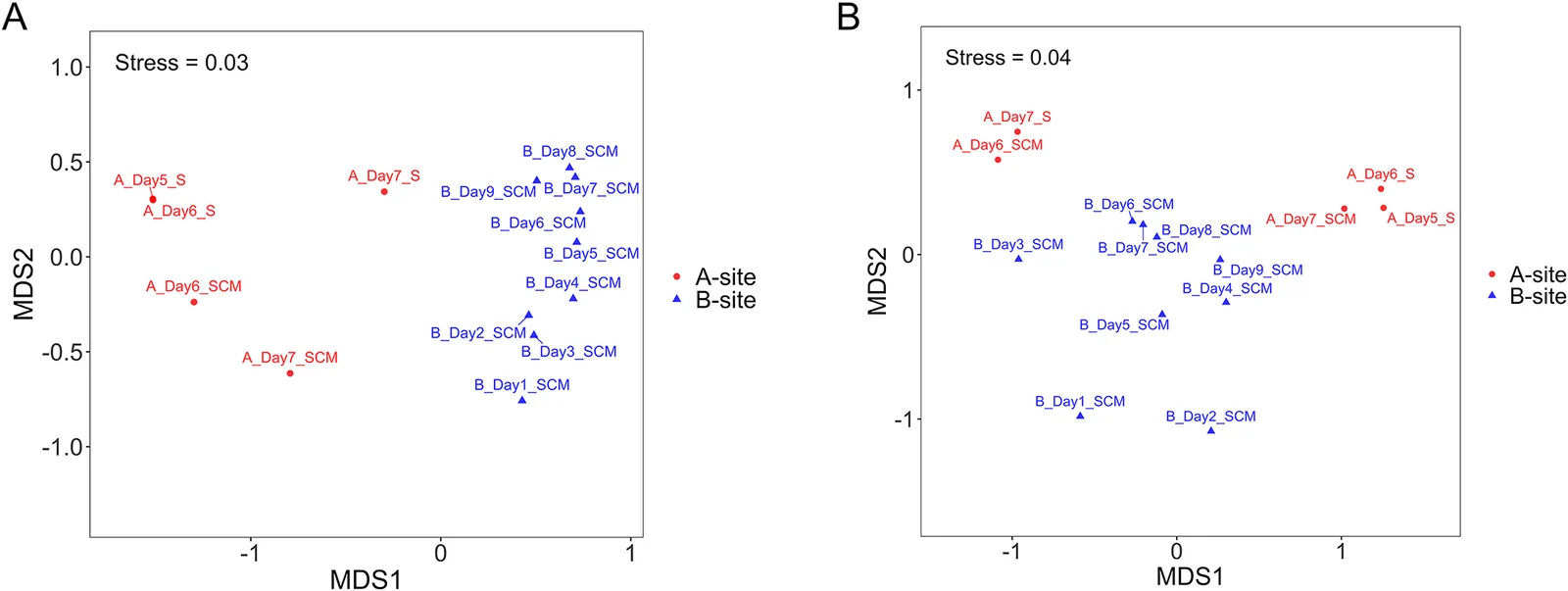
A non-metric multidimensional scaling (NMDS) plot showing the diversity of eukaryotic and giant virus communities based on the Bray-Curtis dissimilarity. (A) Microbial eukaryotic community (18S ASV level, 3- to 144-μm fraction). (B) Giant virus community.

**Fig. 3.**
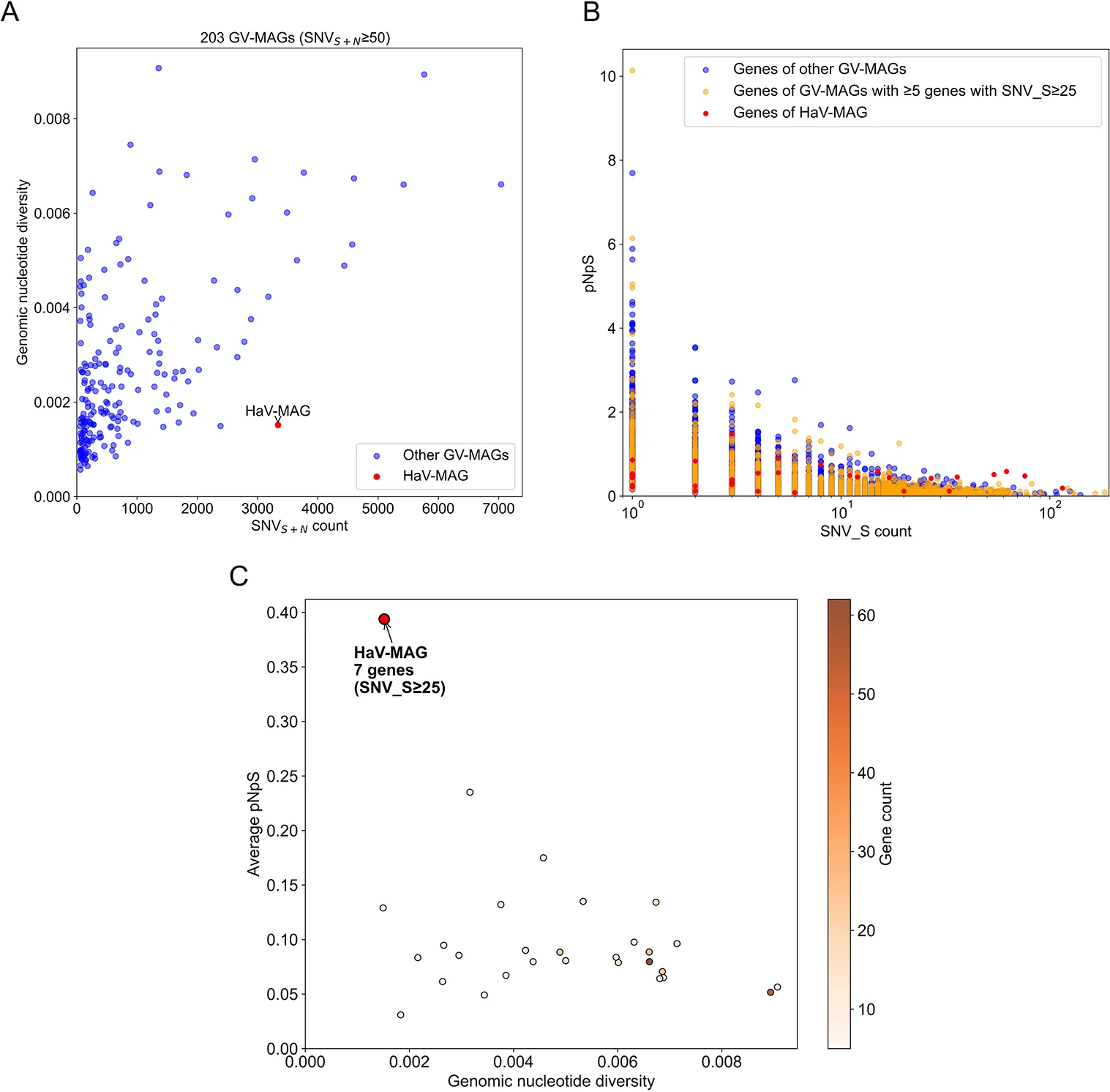
(A) Scatter plot of SNV_S+N_ count—nonsynonymous SNV count across 203 GV-MAGs with SNV_S+N_≥50. Each point represents one GV-MAG; the x-axis indicates the sum of synonymous and nonsynonymous SNVs across all genes in a MAG, and the y-axis shows its genomic nucleotide diversity. (B) Relationship between the synonymous SNV count (SNV_S) and pN/pS at the gene level across 424 GV-MAGs. Genes from GV-MAGs containing ≥5 genes with SNV_S≥25 are shown in orange, genes from HaV-MAG are shown in red, and genes from other GV-MAGs are shown in blue. (C) Scatter plot of genomic nucleotide diversity versus average pN/pS across 29 GV-MAGs, each containing ≥5 genes with SNV_S≥25. Average pN/pS was calculated as the arithmetic mean of pN/pS values for genes with SNV_S≥25. All data were derived from the sample with the maximum relative abundance for each GV-MAG.

**Fig. 4.**
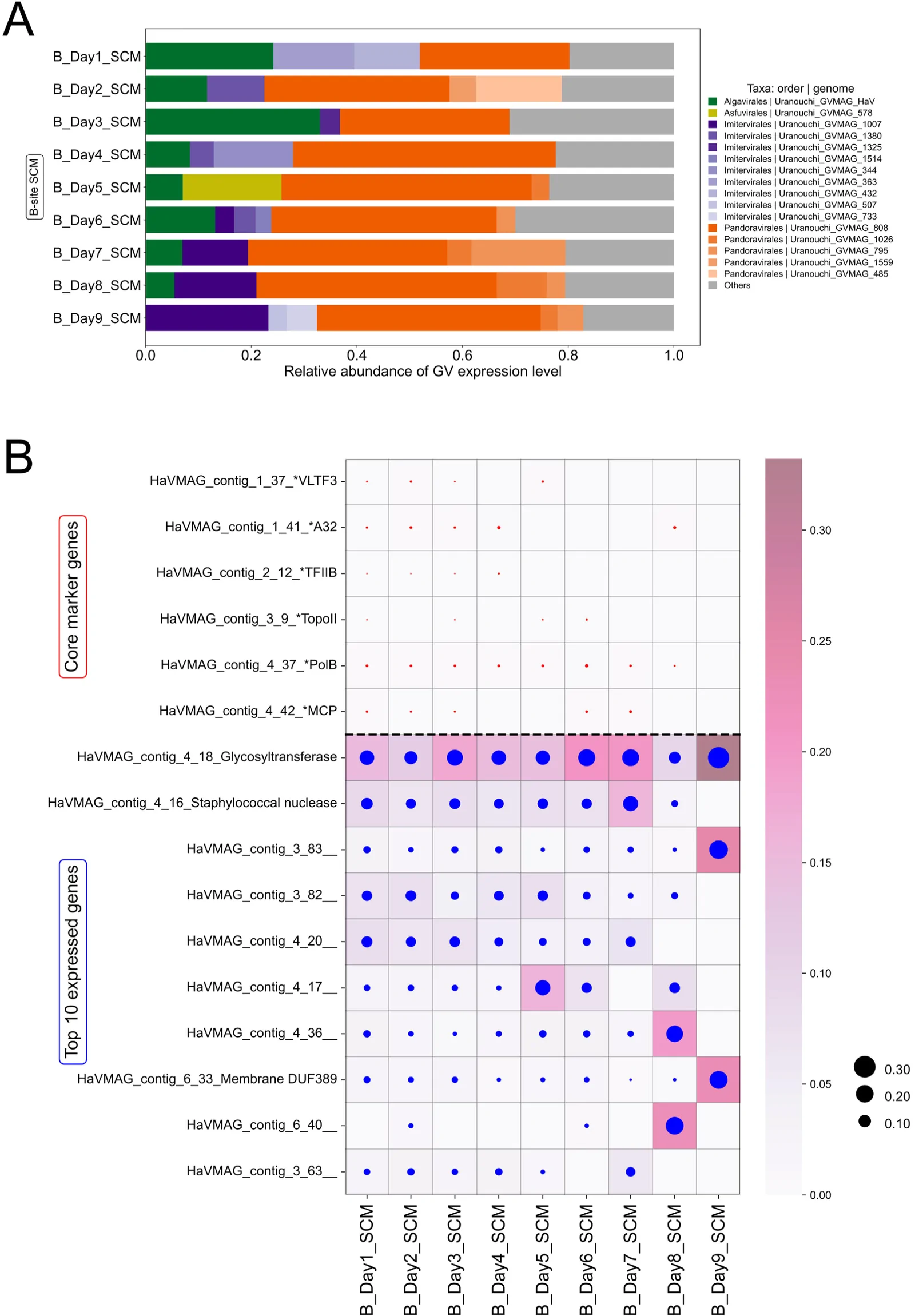
(A) Gene expression level of GV MAGs (normalized to 100% of the GV community, >3% MAGs in at least one sample are listed); (B) HaV gene expression level of core marker genes and top 10 expressed genes (normalized to 100% of HaV genes).

**Table 1.** General features of HaV-MAG and HaV53 reference genome.

Genome	Genome size (bp)	GC content (%)	Number of genes	Number of shared genes	Shared gene percentage	Number of specific genes	ANI
HaV-MAG	258,302	30.0	292	283	96.9%	9	99.86%
HaV53	274,793	30.4	292	272	93.2%	20	
