# Supplementary material for: Rapid Diversification of a Natural Heterosigma akashiwo Virus Population during a Host Bloom
Source: Microbes Environ. 2026 Jul 11;41(3):ME26018. doi: 10.1264/jsme2.ME26018 (PMC13659346; doi:10.1264/jsme2.ME26018)
Supplement: Supplementary file 1 [file 41_26018_s1.pdf]

Supplementary Figures and Text

Rapid diversification of a natural Heterosigma akashiwo virus population during a host bloom

Jun Xia[1], Lingjie Meng[1], Yue Fang[1], Hiroki Ban[1,2], Yusuke Okazaki[1], Takashi Yoshida[3], Hisashi Endo[1], Keizo Nagasaki[4], Hiroyuki Ogata[1]

[1]Institute for Chemical Research, Kyoto University, Gokasho, Uji 611-0011, Japan

[2]Hatsukaichi Field Station, Fisheries Technology Institute, Japan Fisheries Research and Education Agency, Maruishi, Hatsukaichi, Hiroshima 739-0452, Japan

[3]Graduate School of Agriculture, Kyoto University, Kitashirakawa-Oiwake, Sakyo-ku, Kyoto 606-8502, Japan

[4]Faculty of Science and Technology, Kochi University, Nankoku, Kochi 783-8502, Japan

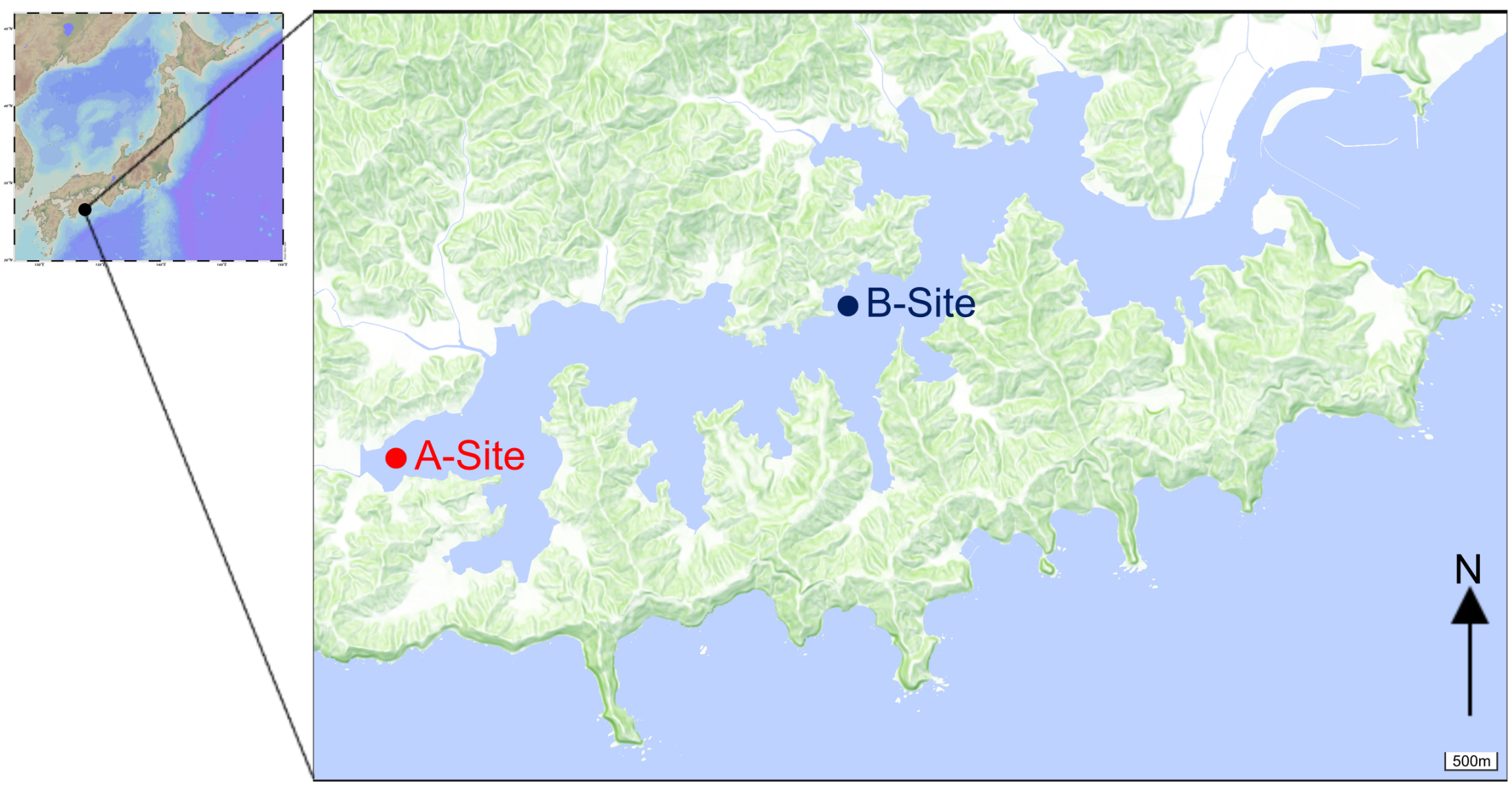

Fig. S1. Location of Uranouchi Bay and the map of sampling sites in this study (A-Site in red tide area of innermost bay [IMB] with red mark, and B-Site closed to a fishery farm of western middle bay [WMB] with deep-blue mark). Base map data are provided by the Ocean Data View (ODV, version 5.7.2) and Geospatial Information Authority of Japan (GSI Maps Vector).

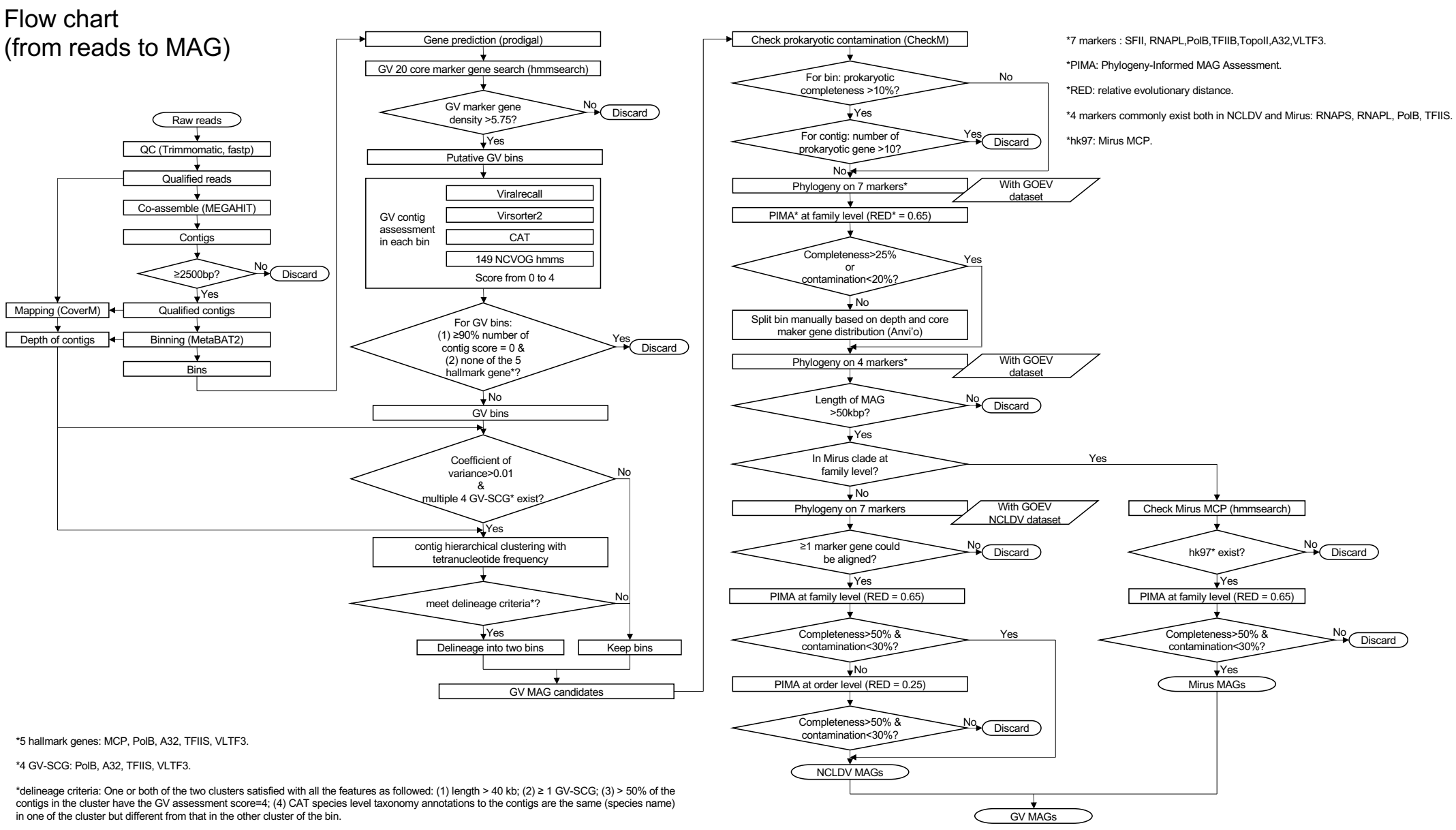

Fig. S2. Flow chart of GV-MAG generating pipeline. (Detailed illustration: see "**Fig. S2 Illustration**" below)

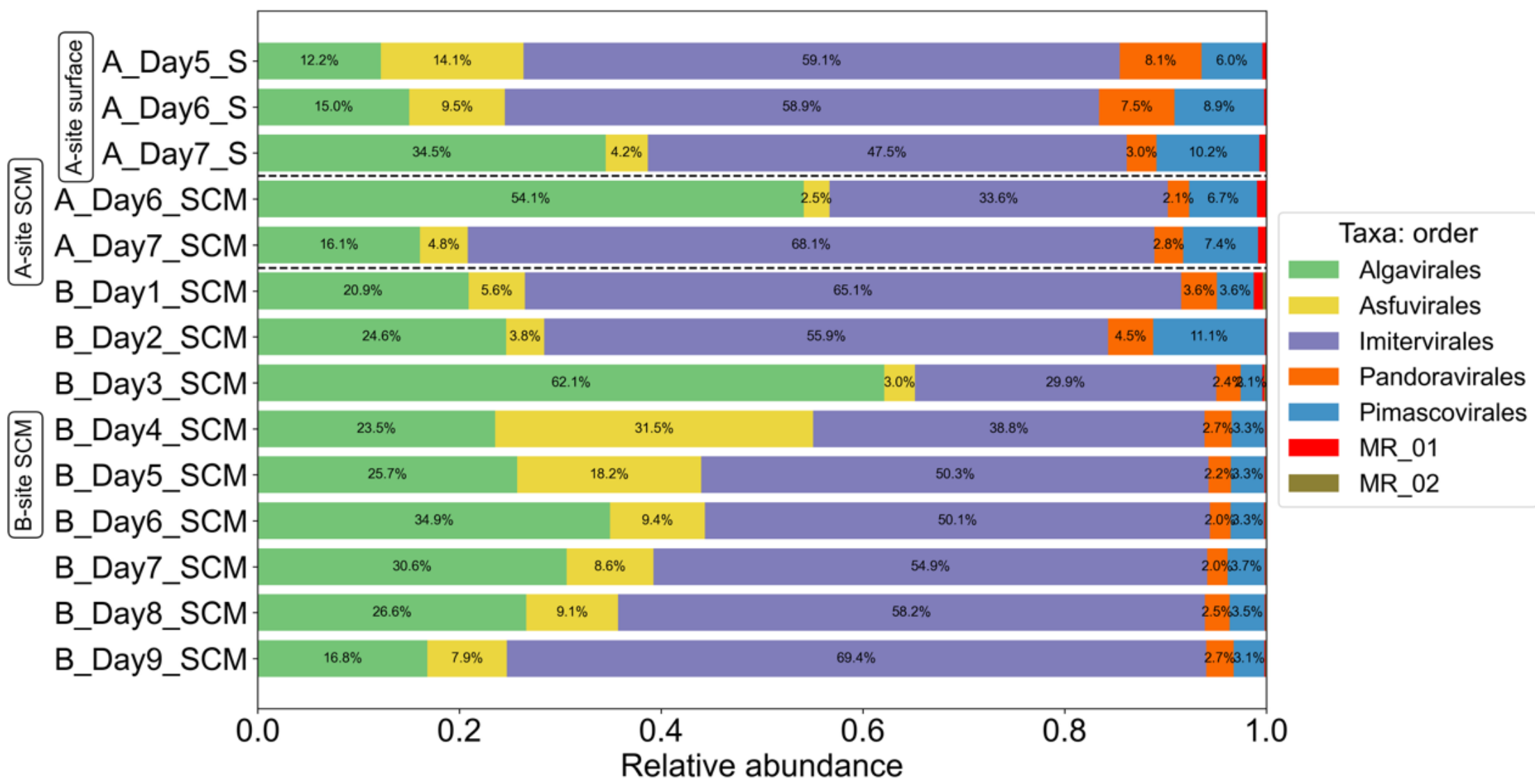

Fig. S3. Relative abundance of GV-MAGs and GOEV genomes in order level in the 14 samples.

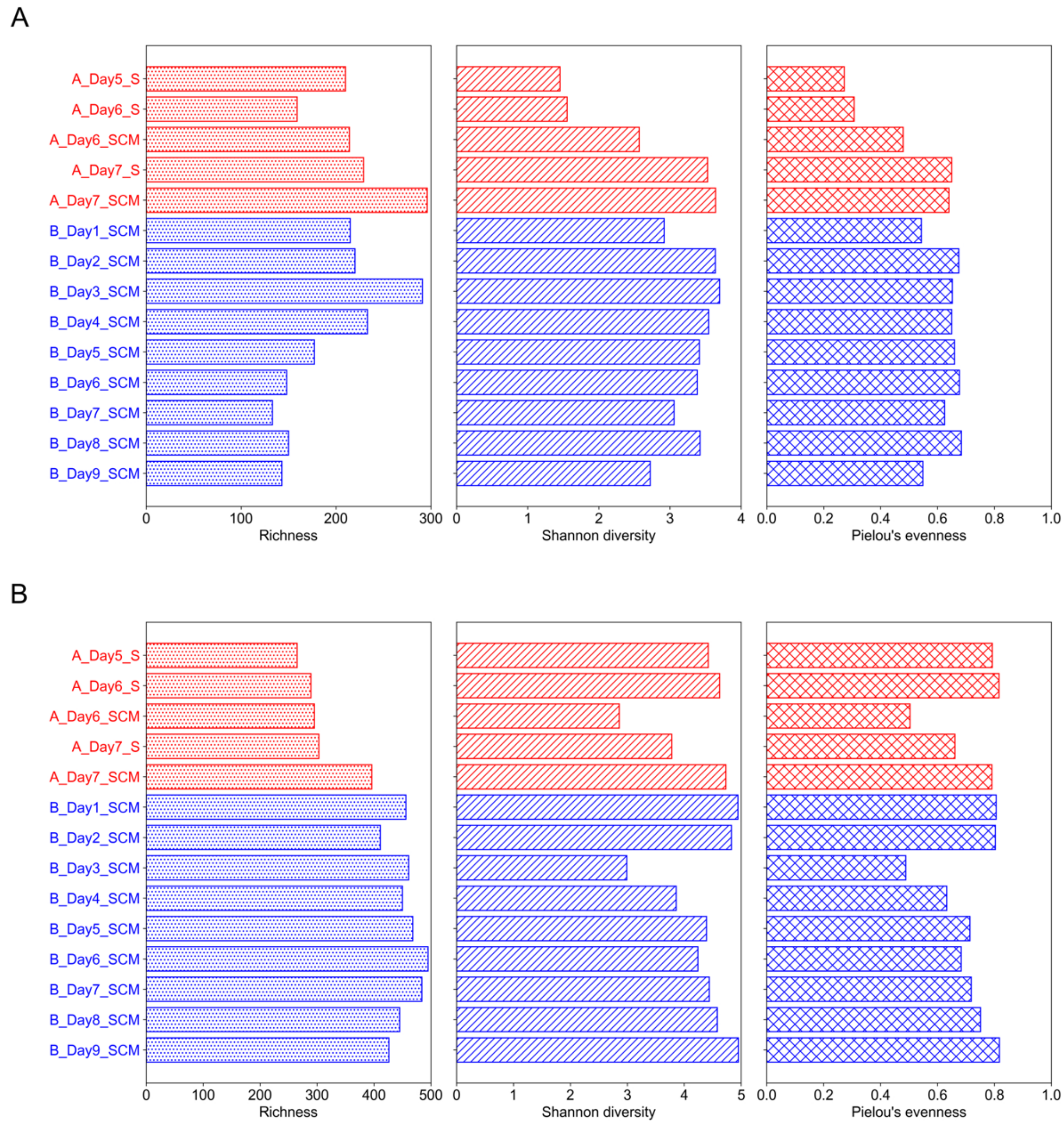

Fig. S4. Richness, Shannon diversity and Pielou's evenness of 14 samples in this study: (A) 3–144 µm microbial eukaryotic 18S community (non-singleton ASVs without metazoa or fungi); (B) 0.22–3 µm giant virus community. S: surface; SCM: Subsurface Chlorophyll Maximum. Lower diversity indices were observed in the Day-5 and Day-6 samples at A-site in the 18S community.

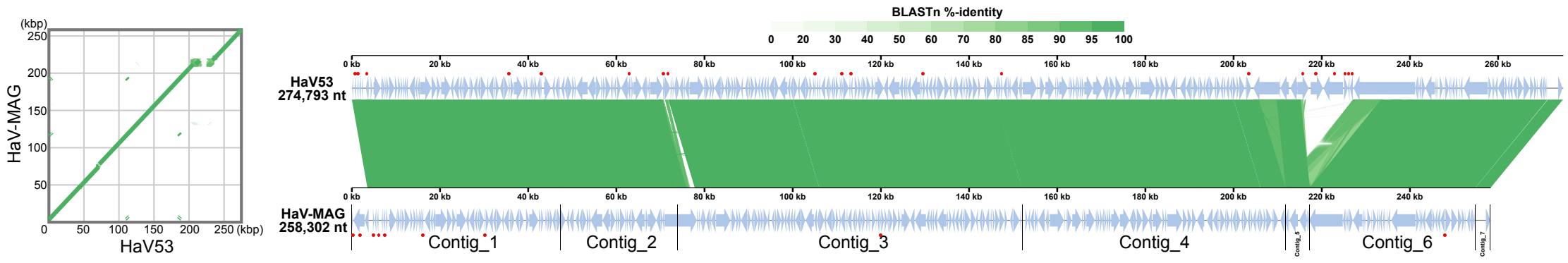

Fig. S5. Left: synteny map of HaV53 and HaV-MAG; right: pairwise gene alignment between HaV53 and HaV-MAG. The nucleotide dissimilar ORFs were marked with red small dot on the right figure (linked to Table S9).

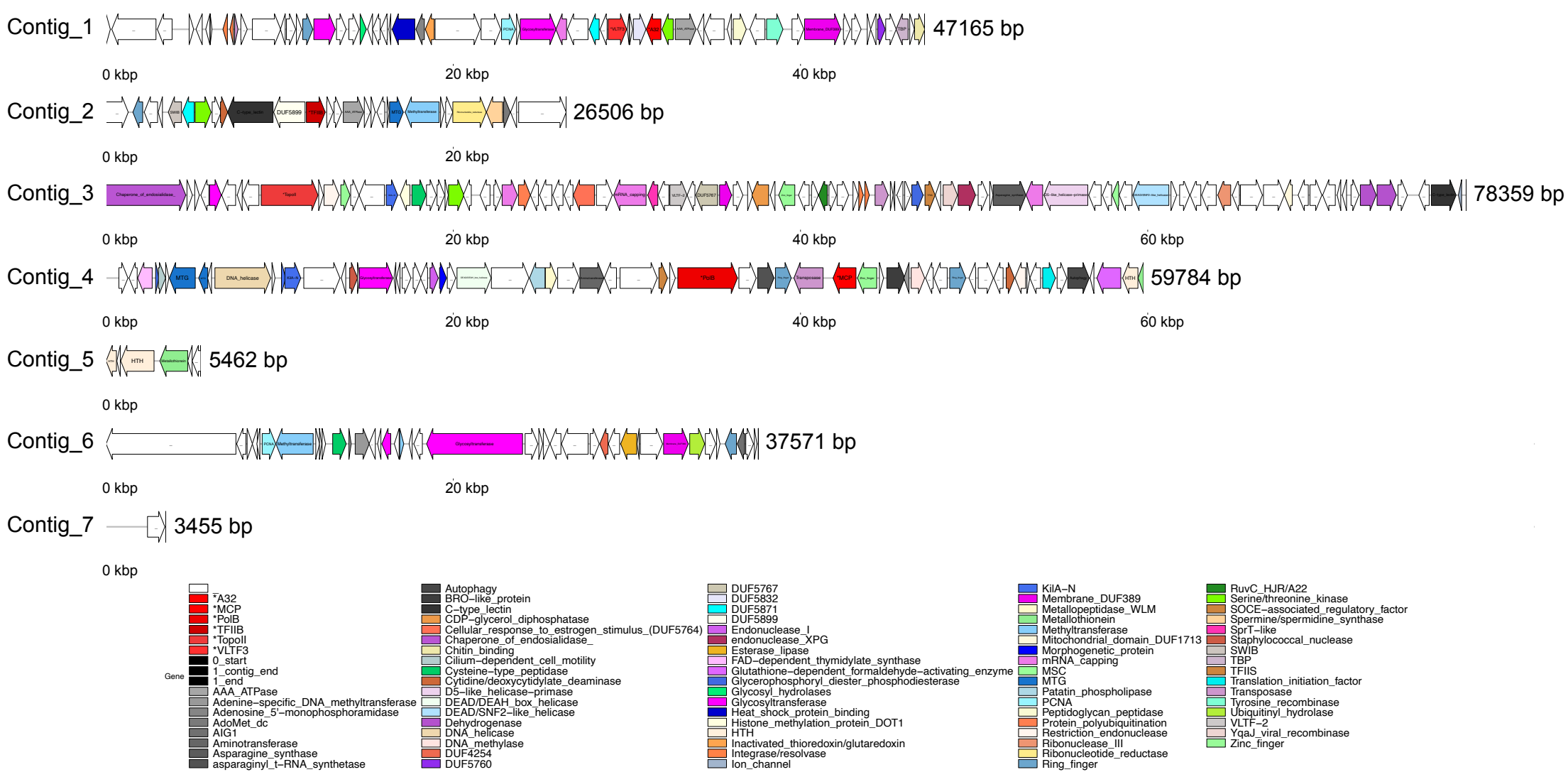

Fig. S6. Genomic structure of HaV-MAG in this study. "*" marked gene refers to one of the nine core marker genes of NCLDV.

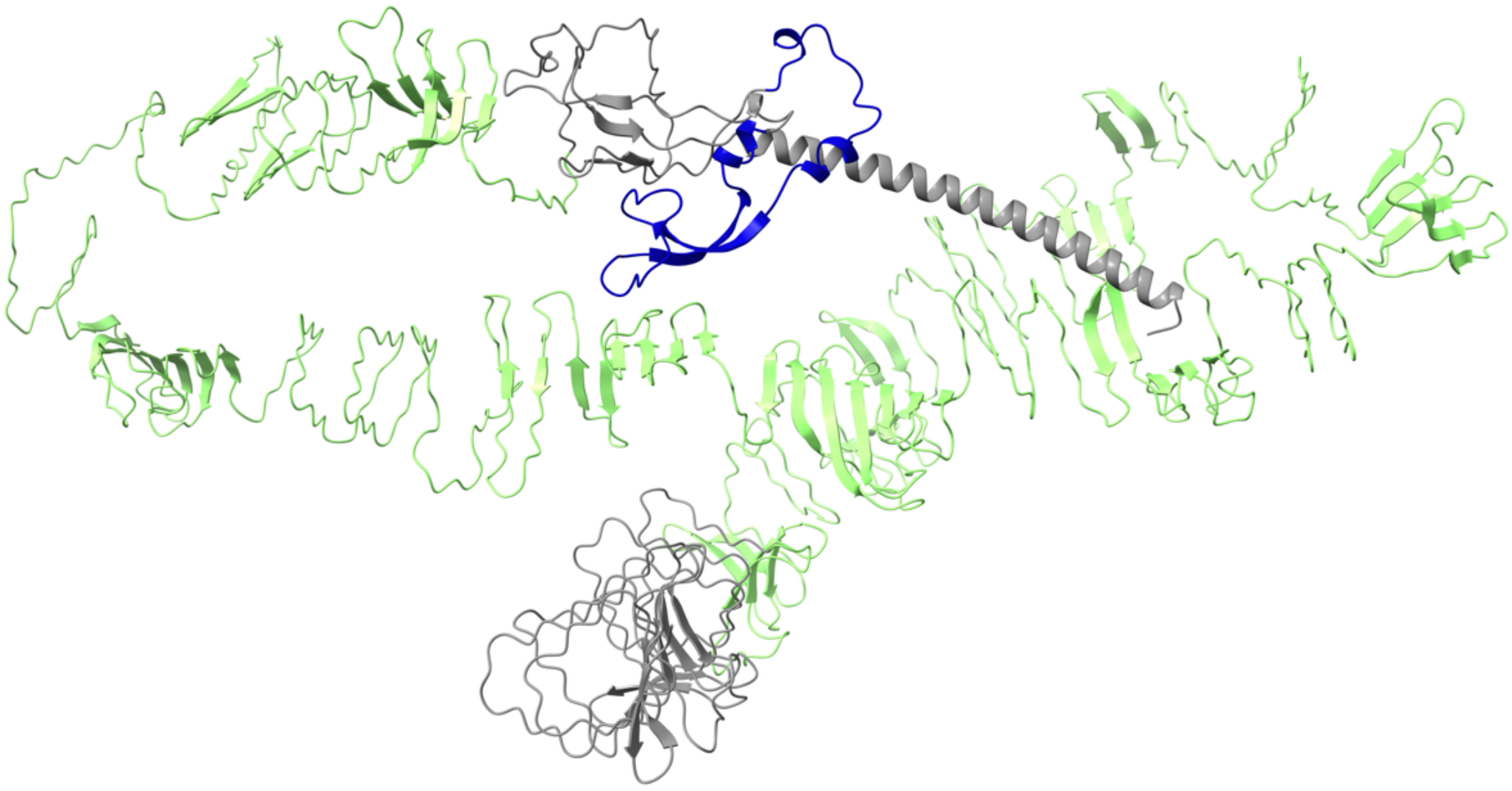

Fig. S7. Protein structure of HaVMAG_contig_3_1 (1530aa) in HaV-MAG. It contains a specific chaperone of endosialidase (deep blue, residues 1380–1439), and a large FhaB domain (light green, residues 217–1329).

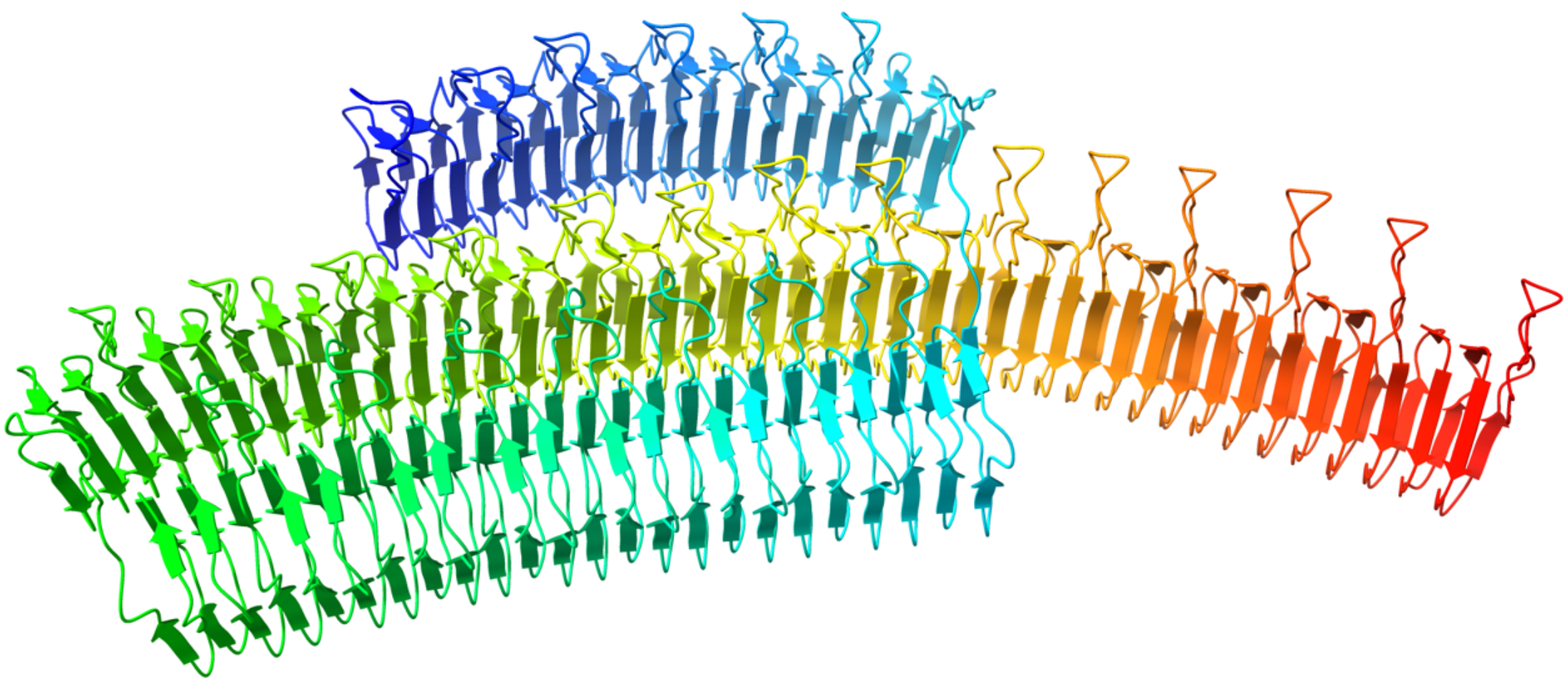

Fig. S8. Partial (from 2580 aa to 4663 aa) structure of the huge protein HaV53_253, the missing repeat-rich region in protein HaVMAG_contig_6_1. N-terminal to C-terminal were colored from red to blue.

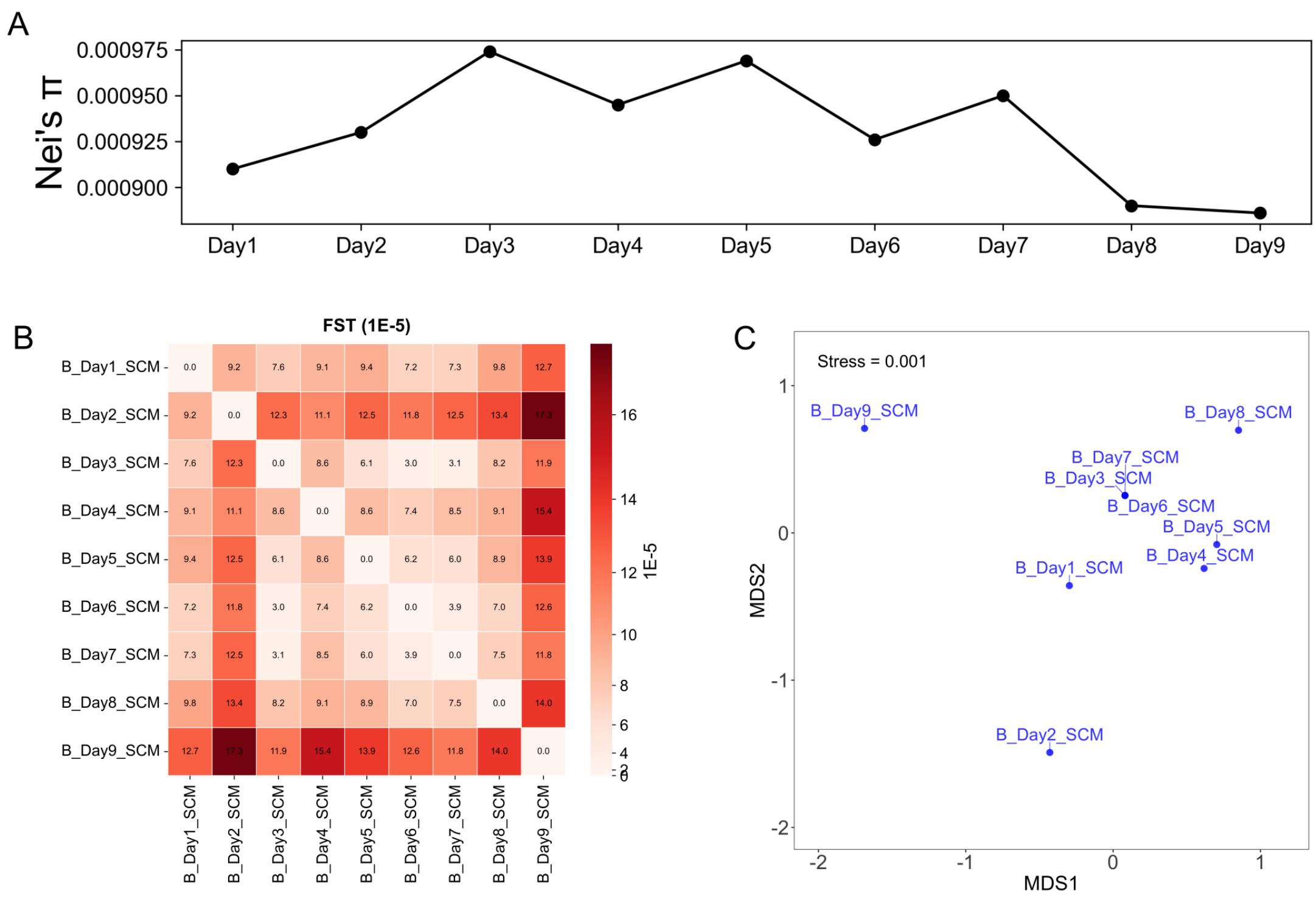

Fig. S9. (A) Nei's nucleotide diversity (Nei's π) of HaV in B-Site during the 9 days. (B) Fixation index (F_{ST}) heatmap of HaV population in B-Site SCM samples, which is from 0 (totally the same) to 1 (totally different). The numerical values displayed in each cell equal the original F_{ST} estimates multiplied by 10^5. Color bar was normalized by "gamma=2.0" to emphasize the high-value grid cells. (C) NMDS of F_{ST} among HaV 9-days population in B-Site.

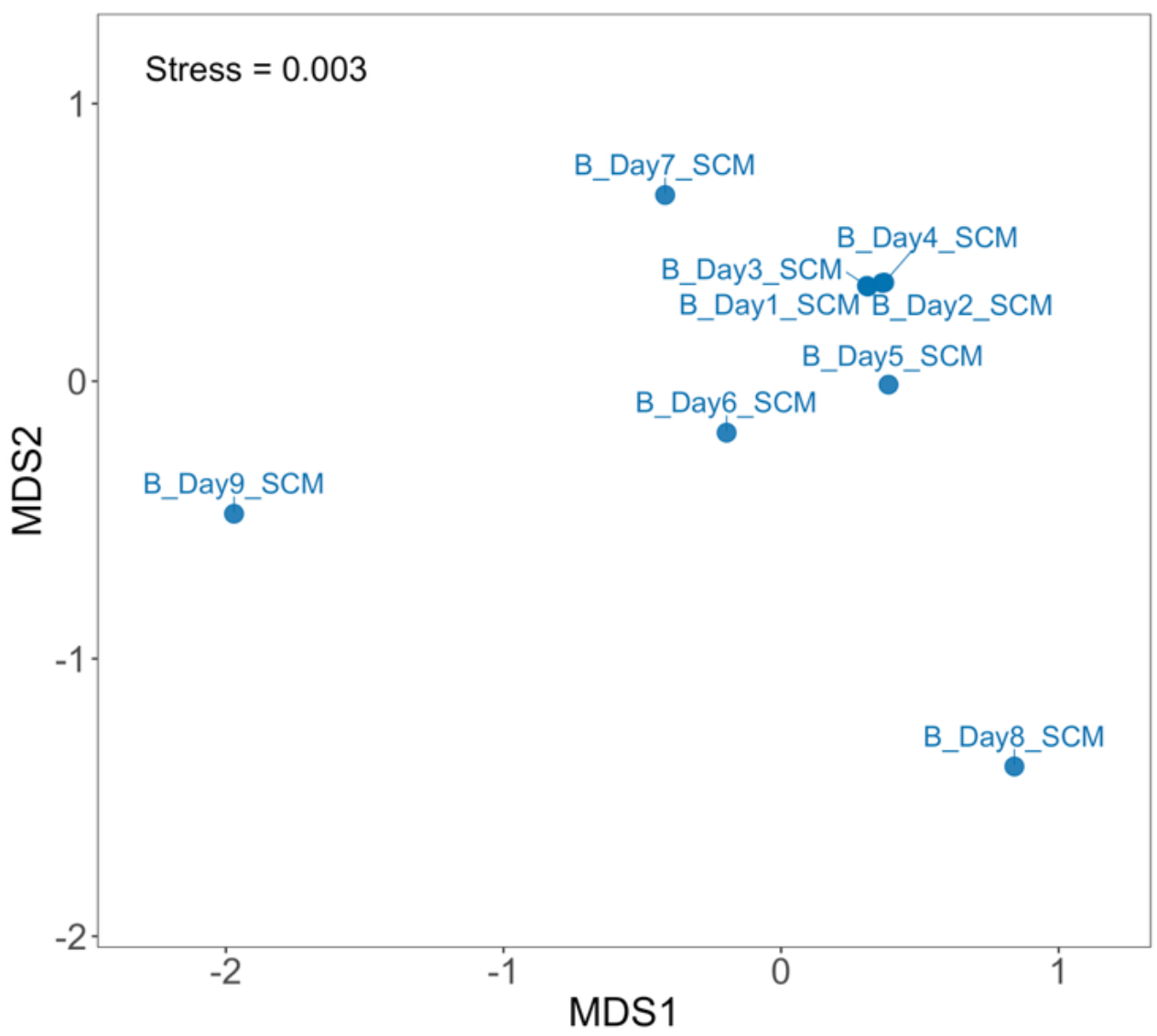

Fig. S10. Dynamics of relative abundance of HaV gene expression level in B-Site 9-day samples.

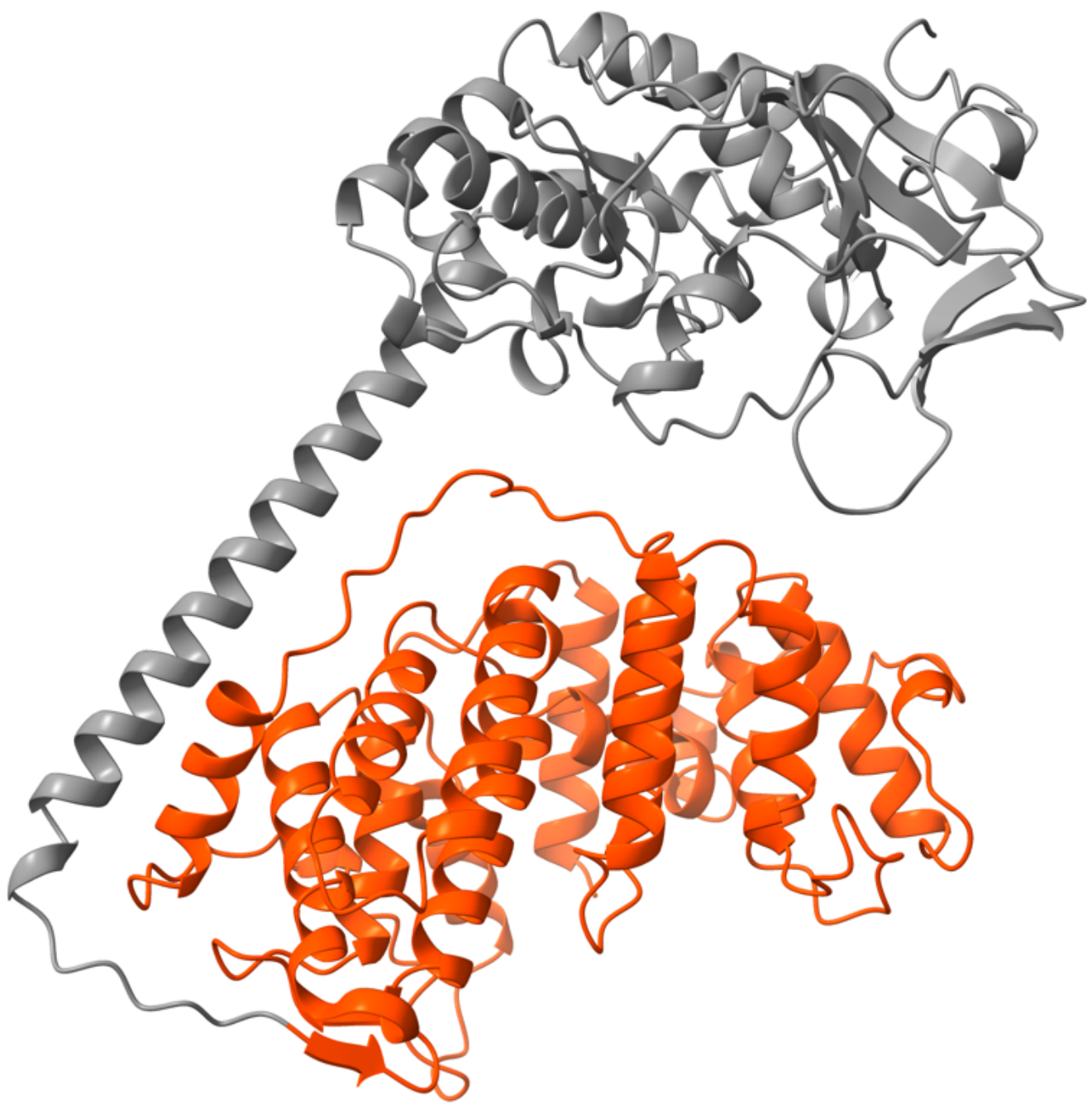

Fig. S11. Protein structure of highly expressed HaV specific glycosyltransferase (GT) in HaV-MAG (HaVMAG_contig_4_18). The α-helices rich region which could define this GT-C fold is highlighted with orange color.

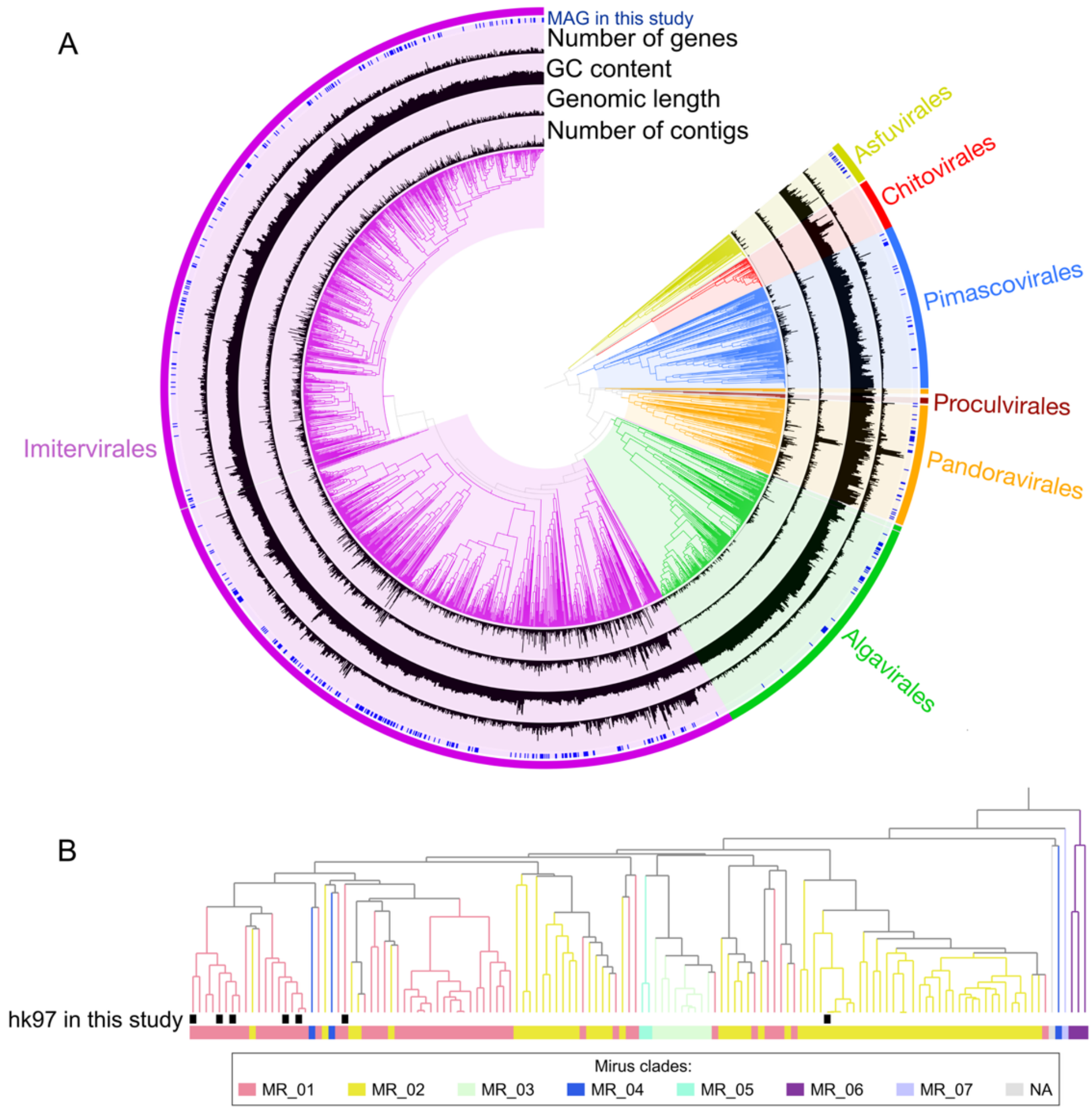

Fig. S12. (A) Maximum-likelihood tree (1,000 bootstraps) based on 7 core marker genes of nucleocytoviruses, rooted by order *Chitovirales* and *Asfuvirales*. 1,692 GOEV nucleocytovirus genomes and 420 Uranouchi nucleocytovirus MAGs were included on the tree. (B) Maximum-likelihood tree (1,000 bootstraps) based on hk97 protein of mirusviruses, rooted by clade MR_06. 136 hk97 proteins (hmmsearch E-value<1e-5) from 77 GOEV mirusvirus MAGs and 4 Uranouchi mirusvirus MAGs were included on the tree. To facilitate unambiguous visualization of taxonomic relationships, all branches of the phylogeny were normalized to a uniform maximum evolutionary distance.

Fig. S2 Illustration

PART I: reads QC, assemble, initial binning. Reads quality control was performed by Trimmomatic and fastp, then co-assembled by MEGAHIT for samples in both size-fractions. Contigs with length more than 2500 bp were kept for downstream analysis. Depth of each contigs were calculated based on qualified reads mapping on contigs by CoverM (v0.7.0) (Aroney *et al*., 2025). Binning was performed by MetaBAT2 (v2.15) (Kang *et a*l., 2019).

PART II: selection of GV-MAG candidates. Predicted ORFs (by prodigal [v2.6.3] [Hyatt *et al*., 2010]) in total bins were matched on 20 core marker gene Hidden Markov models (hmms) of giant viruses by hmmsearch. Bins with GV core gene density over 5.75 were kept as putative GV bins (Fang *et al*., 2025). GV contig assessment was performed sequentially by ViralRecall (v2.1) (Aylward and Moniruzzaman, 2021), VirSorter2 (v2.2.3) (Guo *et al*., 2021), CAT database (v2021-01-07) (von Meijenfeldt *et al*., 2019), 149 NCVOG hmms (Yutin *et al*., 2009; Gaïa *et al*., 2023). Each passed step will make the bin score "+1". A bin was treated as unqualified when both two conditions met: (1) over 90% number of contigs scored as 0; (2) none of the five hallmark genes (MCP, PolB, TFIIB, TopoII, and A32) found. While depth coefficient of variation >0.01 and multiple copies of 4 GV-SCGs exist, a bin was further delineaged into two bins based on tetranucleotide frequency and depth.

PART III: GV-MAG refinement. Prokaryotic completeness in each bin was calculated by CheckM (v1.2.2) (Parks *et al*., 2015). If contamination >10% and number of prokaryotic gene >10 in a bin or concentrated in a contig, it was discarded. NCLDV

or Mirus core marker gene sets in protein format were searched, concatenate-aligned and trimmed by ncldv_markersearch.py (v1.1) (Aylward *et al*., 2021), followed with phylogenetic analysis by iqtree2 (v2.2.2.6) (Minh *et al*., 2020). Giant virus genome completeness and contamination were evaluated by Phylogeny-informed MAG assessment (PIMA) (Fang *et al.*, 2025). The bins with completeness >25%, contamination <20% and size >50 kbp were then kept. MAG refinement based on contig depth Dendrogram was manually performed by Anvi'o (v7.1) (Eren *et al*., 2015). Final NCLDV MAGs should be qualified if (1) on the 7 core marker gene tree; (2) completeness >50% and contamination <30% at both family level (RED>0.65) and order level (0.25<RED≤0.65). For recognizing Mirus features, conditions below should be satisfied in a MAG: (1) contained in Mirus families; (2) Mirus-specific major capsid protein hk97 (Gaïa *et al*., 2023) detected (hmmsearch [v3.4] [Finn *et al*., 2011], e-value<1e-5); (3) completeness >50% and contamination <30%.

Giant virus MAGs recovery and taxonomic assignment

Our computational pipeline and manual curation yielded 424 high-quality GV-MAGs with genome lengths ranging from 59.3 kbp up to 1.36 Mbp (see Materials and Methods, Fig. S2 and Fig. S2 Illustration). These MAGs were classified in either *Nucleocytoviricota* (420 GV-MAGs) or *Mirusviricota* (4 GV-MAGs). One MAG (HaV-MAG after manual curation) showed 99.86% ANI with the reference HaV53 genome. A phylogeny of nucleocytoviruses based on the core genes together with 1,692 genomes from GOEV assigned the 420 nucleocytovirus MAGs to *Imitervirales* (N=319),

Algavirales (N=37), *Pandoravirales* (N=30), *Pimascovirales* (N=19), or *Asfuvirales* (N=14) (Fig. S12A). One nuclecytovirus MAG formed a long branch in the tree near the *Pandoravirales* clade and could not be assigned to a known order. A mirusvirus phylogenetic tree based on HK97 sequences together with those from 84 GOEV mirusvirus genomes assigned three mirusvirus MAGs to clade MR_01 and one to clade MR_02 (Fig. S12B). Except HaV-MAG, none of other 423 GV-MAGs shared >95% ANI with known reference viral genomes. When compared with previously published GV-MAGs (*i.e.*, GOEV MAGs), additional 19 nuclecytovirus MAGs and one mirusvirus MAG produced in this study shared >95% ANI with GOEV MAGs (Table S7). It should be noted that, even when compared with the 1,065 GV-MAGs recovered from long-term sampling in Uranouchi Inlet (Fang *et al*., 2025), more than half of the 424 GV-MAGs identified in this study were missed in the seasonal sampling. Only 169 of 424 GV-MAGs exhibited ≥95% ANI with at least one of the 1,065 previously reported GV-MAGs.

Environmental parameters and measurement methods

Metadata include temperature, salinity, dissolved oxygen (DO), Chlorophyll-*a* and depth were detected by sensors of YSI Multiparameter Digital Water Quality Meter (Xylem Inc.) in situ. The chlorophyll sensor was lowered down gradually from the surface to a depth of approximately 10 m while continuously monitoring chlorophyll concentration in real time, the depth at which reach to a maximum was defined as Depth of Subsurface Chlorophyll Maximum (SCM). Samples for nutrient analysis were collected in 10 mL acrylic tubes and stored at –20°C until analysis. Samples for

particulate organic carbon (POC) were collected onto pre-combusted (450°C, 4h) 47 mm GF/F filters (Whatman) with a gentle vacuum (<0.013 MPa), and the filter samples were stored at –20°C until analysis. The filtrates were collected in glass vials for dissolved organic carbon (DOC) analysis and stored at –20°C until analysis. Concentrations of POC were analyzed using a Sumigraph NC analyzer (NC-22F, Sumika). Prior to analysis, the filter samples were exposed to HCl fumes to remove inorganic carbon and then completely dried in a desiccator under vacuum for 24h. DOC was measured the high-temperature combustion method using a total organic carbon analyzer (TOC-V CSH, Shimadzu). Nutrient concentrations (nitrate + nitrite, nitrite, ammonium, phosphate, and silicate) were measured by an autoanalyzer (Bran + Luebbe Autoanalyzers) (Yasunaka *et al*., 2018).

Environmental parameters were measured for all samples at B-site (from B_Day1_SCM to B_Day9_SCM), and part of A-site (A_Day5_S, A_Day7_S and A_Day7_SCM) (Table S1). Seawater temperature ranged from 22.28°C to 24.20°C. Chlorophyll *a* (Chl-*a*) concentrations fluctuated over nine days in the SCM layer at B-site (13.21-78.36 μg/mL). It exceeded the detection limit (99.99 μg/mL) at the surface of A-site on Day-5, and declined on Day-7 (2.91 μg/mL at surface, 15.36 μg/mL at SCM). Chl-*a* concentration was positively correlated with normalized DO, phosphate, and particulate organic carbon (POC) concentrations (Pearson's correlation, adjusted $p < 0.05$; Table S2).

References

Aroney, S. T., Newell, R. J., Nissen, J. N., Camargo, A. P., Tyson, G. W., and Woodcroft, B. J. (2025) CoverM: Read alignment statistics for metagenomics. *Bioinformatics* **41**: btaf147.

Aylward, F. O., and Moniruzzaman, M. (2021) ViralRecall—A flexible command-line tool for the detection of giant virus signatures in 'omic data. *Viruses* **13**: 150.

Aylward, F. O., Moniruzzaman, M., Ha, A. D., and Koonin, E. V. (2021) A phylogenomic framework for charting the diversity and evolution of giant viruses. *PLoS Biol* **19**: e3001430.

Eren, A. M., Esen, Ö. C., Quince, C., Vineis, J. H., Morrison, H. G., Sogin, M. L., *et al.* (2015) Anvi'o: an advanced analysis and visualization platform for 'omics data. *PeerJ* **3**: e1319.

Fang, Y., Meng, L., Xia, J., Gotoh, Y., Hayashi, T., Nagasaki, K., Endo, H., Okazaki, Y., *et al.* (2025) Genome-resolved year-round dynamics reveal a broad range of giant virus microdiversity. *mSystems* **10**: e01168-24.

Finn, R. D., Clements, J., and Eddy, S. R. (2011) HMMER web server: interactive sequence similarity searching. *Nucleic Acids Res* **39**: W29–W37.

Gaïa, M., Meng, L., Pelletier, E., Forterre, P., Vanni, C., Fernandez-Guerra, A., *et al.* (2023) Mirusviruses link herpesviruses to giant viruses. *Nature* **616**: 783–789.

Guo, J., Bolduc, B., Zayed, A. A., Varsani, A., Dominguez-Huerta, G., Delmont, T. O., *et al.* (2021) VirSorter2: a multi-classifier, expert-guided approach to detect diverse DNA and RNA viruses. *Microbiome* **9**: 37.

Hyatt, D., Chen, G.-L., LoCascio, P. F., Land, M. L., Larimer, F. W., and Hauser, L. J. (2010) Prodigal: prokaryotic gene recognition and translation initiation site identification. *BMC Bioinf* **11**: 119.

Kang, D. D., Li, F., Kirton, E., Thomas, A., Egan, R., An, H., *et al.* (2019) MetaBAT 2: an adaptive binning algorithm for robust and efficient genome reconstruction from metagenome assemblies. *PeerJ* **7**: e7359.

Minh, B. Q., Schmidt, H. A., Chernomor, O., Schrempf, D., Woodhams, M. D., von Haeseler, A., *et al.* (2020) IQ-TREE 2: New models and efficient methods for phylogenetic inference in the genomic era. *Mol Biol Evol* **37**: 1530–1534.

Parks, D. H., Imelfort, M., Skennerton, C. T., Hugenholtz, P., and Tyson, G. W. (2015) CheckM: assessing the quality of microbial genomes recovered from isolates, single cells, and metagenomes. *Genome Res* **25**: 1043–1055.

von Meijenfeldt, F. A. B., Arkhipova, K., Cambuy, D. D., Coutinho, F. H., and Dutilh, B. E. (2019) Robust taxonomic classification of uncharted microbial sequences and bins with CAT and BAT. *Genome Biol* **20**: 217.

Yasunaka, S., Nojiri, Y., Hashioka, T., Yoshikawa, C., Kodama, T., Nakaoka, S., *et al.* (2018) Basin-scale distribution of NH_4^+ and NO_2^- in the Pacific Ocean. *J Oceanogr* **74**: 1–11.

Yutin, N., Wolf, Y. I., Raoult, D., and Koonin, E. V. (2009) Eukaryotic large nucleo-cytoplasmic DNA viruses: clusters of orthologous genes and reconstruction of viral genome evolution. *Virol J* **6**: 223.
